# Evaluation of Myocilin Variant Protein Structures Modeled by AlphaFold2

**DOI:** 10.3390/biom14010014

**Published:** 2023-12-21

**Authors:** Tsz Kin Ng, Jie Ji, Qingping Liu, Yao Yao, Wen-Ying Wang, Yingjie Cao, Chong-Bo Chen, Jian-Wei Lin, Geng Dong, Ling-Ping Cen, Chukai Huang, Mingzhi Zhang

**Affiliations:** 1 Joint Shantou International Eye Center of Shantou University and The Chinese University of Hong Kong, Shantou 515041, China; micntk@hotmail.com (T.K.N.);; 2 Department of Ophthalmology and Visual Sciences, The Chinese University of Hong Kong, Hong Kong, China; 3 Network & Information Centre, Shantou University, Shantou 515041, China; 4 Key Laboratory of Carbohydrate and Lipid Metabolism Research, College of Life Science and Technology, Dalian University, Dalian 116622, China; 5 Shantou University Medical College, Shantou 515041, China

**Keywords:** AlphaFold2, myocilin, variants, protein structure, molecular simulation

## Abstract

Deep neural network-based programs can be applied to protein structure modeling by inputting amino acid sequences. Here, we aimed to evaluate the AlphaFold2-modeled myocilin wild-type and variant protein structures and compare to the experimentally determined protein structures. Molecular dynamic and ligand binding properties of the experimentally determined and AlphaFold2-modeled protein structures were also analyzed. AlphaFold2-modeled myocilin variant protein structures showed high similarities in overall structure to the experimentally determined mutant protein structures, but the orientations and geometries of amino acid side chains were slightly different. The olfactomedin-like domain of the modeled missense variant protein structures showed fewer folding changes than the nonsense variant when compared to the predicted wild-type protein structure. Differences were also observed in molecular dynamics and ligand binding sites between the AlphaFold2-modeled and experimentally determined structures as well as between the wild-type and variant structures. In summary, the folding of the AlphaFold2-modeled MYOC variant protein structures could be similar to that determined by the experiments but with differences in amino acid side chain orientations and geometries. Careful comparisons with experimentally determined structures are needed before the applications of the in silico modeled variant protein structures.

## 1. Introduction

Glaucoma is a leading cause of irreversible visual impairment and blindness worldwide. Primary open-angle glaucoma (POAG), a major type of glaucoma, affects more than 65 million individuals globally [1]. Current clinical treatments for POAG are mainly based on intraocular pressure-lowering medications and surgeries [2]. Exploring new molecular-targeted therapies is urgently needed. Human myocilin (*MYOC*) gene, located on chromosome 1q24.3–1q25.2 [3], is the first disease-causing gene identified for POAG by the family-linkage analysis [4], accounting for 27% familial and 2% sporadic juvenile-onset POAG (JOAG) cases [5]. *MYOC* encodes for a secretory glycoprotein with 504 amino acids [6], composed of a signal sequence (residue 1–32), the N-terminal coiled-coil domain with the leucine zipper motifs (residue 33–201), the intermediate linker region (residue 202–243), and the C-terminal olfactomedin-like domain (residue 244–504) with an N-linked glycosylation at N57-E58-S59 and a disulfide bond linking C245 and C433 [7]. MYOC protein can be cleaved by CAPN2, an intracellular calcium-dependent protease localized in the endoplasmic reticulum [8], between R226 and I227, separating the C-terminal olfactomedin-like domain fragment and the N-terminal coiled-coil domain [9]. Although the complete crystal structure of the full-length human myocilin protein still remains unsolved, the crystal structures of a part of the N-terminal coiled-coil domain of mouse MYOC protein (PDB ID: 5VR2) [10] and the complete C-terminal olfactomedin-like domain of human MYOC protein (PDB ID: 4WXQ) have been resolved [11] in that the olfactomedin-like domain was observed to be a five-bladed β-propeller, whereas the N-terminal coiled-coil domain suggests a Y-shaped α-helical parallel dimer. Notably, the experimentally determined MYOC variant protein structures are limited. Resolving the MYOC variant protein structures can facilitate the development of target drugs against MYOC mutation-associated POAG.

At present, more than 100 *MYOC* variants have been identified [12], and more than 90% of the disease-causing *MYOC* variants were located in the olfactomedin-like domain [13]. Among the disease-causing *MYOC* variants, the p.Q368* (c.1102C>T) variant is the most common *MYOC* risk variant for glaucoma among individuals of European ancestry with the prevalence highest in Finland [14]. Determination of the pathogenicity of genetic variants is challenging without the clinical and experimental information [15]. Apart from eliminating the potential of the disease-causing variants by their presence in the control subjects [16], bioinformatics programs, such as Polyphen [17], SIFT [18], MutationTaster [19], and CADD [20], have been developed to assist the prediction of gene variant pathogenicity. However, without the visualization of the protein structure, these programs hardly provide precise structural and conformational changes for the pathogenicity determination. Compared to changing the amino acid residues on the experimentally determined protein structures by the protein structure visualization tools, variant protein structure modeling should potentially assist the evaluation of the pathogenicity of genetic variants by understanding the structural and conformational changes in the variant protein.

Protein structures can be determined experimentally by X-ray crystallography [21], nuclear magnetic resonance (NMR) spectroscopy [22], and cryo-electron microscopy [23]. The explosion of the human genome project creates a gap between the number of identified protein sequences and experimentally resolved protein structures but induce an eagerness to utilize computational modeling (template-based modeling and template-free modeling) strategies for protein structure prediction [24]. With the advancement in artificial intelligence, deep neural network-based programs, including AlphaFold [25] and RoseTTAFold [26], can now be used to model protein structures by inputting the amino acid sequence of a protein. Based on the idea from Anfinsen that the information encoded in the amino acid sequence of a protein determines its structure [27], we herein aimed to evaluate whether the deep neural network-based protein structure modeling programs could construct respective variant protein structures based on the amino acid sequences of the variant proteins. In this study, we compared the structure similarity and amino acid side chain geometry of the MYOC variant protein structures modeled by AlphaFold2 to the experimentally determined structures from the protein data bank (PDB) [28]. The modeled protein structures were further analyzed by the AMBER molecular dynamic (MD) [29] and Schrödinger molecular docking programs [30].

## 2. Materials and Methods

### 2.1. Protein Structure Modeling by AlphaFold2

The open-source version of Alphafold2 was released by DeepMind on 15 July 2021 [25]. The recommended running environment is based on Docker. The codebase of Alphafold2 (version 2.0) was downloaded to our servers on 10 August 2021 and built using Cuda 11.1, with some modifications to the parameters and source code. The Alphafold2 neural network parameters and related databases were downloaded from 10 to 26 August 2021. Considering the performance of computer hardware, in order to improve computing speed, the n_cpu parameters in the data/tools/hhblits.py and data/tools/jackhammer.py files were set to be doubled. The following two lines were added into the pipeline.py script to fix a bug (http://alphafold.hegelab.org/, accessed on 11 December 2021).

uniref90_msa = uniref90_msa[:self.uniref_max_hits] # hege

uniref90_deletion_matrix = uniref90_deletion_matrix[:self.uniref_max_hits] # hege

It should be mentioned that Alphafold2 continues evolving, and the latest version is 2.1.1, especially since some APIs have been changed. The above method might only be suitable for the specific version.

AlphaFold2 is equipped with a prediction script ‘run_docker.py’ and a command line example that calls this script. A custom program was developed to predict the sequences in parallel using different GPUs and automatically using different parameters for different sequences. The parameter ‘preset’ was set to ‘reduced_dbs’ for sequences longer than 2000 and ‘full_dbs’ for others, and the parameter ‘max_template_date’ was set to ‘2021-08-14′, which was prior to the dates of protein structures that were used for the validation of the experimentally determined structures obtained from PDB. Five structures were obtained for each input sequence. After performing an amber15 relaxation procedure on the unrelaxed structure prediction, Alphafold produces a per-residue estimate of its confidence (pLDDT) on a scale from 0 to 100 corresponding to the model’s predicted score on the IDDT-Cα metric. The five predicted structures were ordered according to the pLDDT score, and Rank 0 structure was the prediction with the highest pLDDT score.

The amino acid sequences input into the AlphaFold2 for protein structure modeling were retrieved from the PDB and the National Center for Biotechnology Information database (https://www.ncbi.nlm.nih.gov/protein, accessed on 11 December 2021). The amino acid sequence of human *MYOC* transcript (ID: NM_000261.2), without the signal peptide sequence (1–32 residues), was adopted in the AlphaFold2 modeling of wild-type and variant MYOC protein structures.

### 2.2. Protein Structures from Protein Data Bank

In total, 10 experimentally determined variant protein structures (PDB ID: 6SSO, 7AHF, 7JZ7, 7K1A, 7K77, 7LCA, 7LCL, 7N4X, 7RLG, and 7S2N), which were released after 1 September 2021, were randomly retrieved from the PDB to validate the variant protein structure modeling potential of AlphaFold2. Moreover, 8 experimentally determined structures for human wild-type (PDB ID: 4WXQ) and variant MYOC protein (PDB ID: 4WXS (p.E396D), 6OU2 (p.D478N), 6OU3 (p.D478S), 6OU0 (p.D380A/p.D478S), 6PKD (p.N428D/p.D478H), 6PKE (p.N428E/p.D478S), and 6PKF (p.N428E/p.D478K) [11,31,32] were also retrieved from the PDB for the comparison analysis with the AlphaFold2-modeled structures.

### 2.3. Structure Similarity Analyses

The protein structures were visualized and aligned by PyMOL (https://www.pymol.org, accessed on 11 December 2021). The similarity of the protein structures was evaluated by the template modeling (TM) score [33], the local Distance Difference Test (lDDT) score [34], and the overall root-mean-square deviation (RMSD) by local–global alignment (LGA) analysis [35] with default settings. TM-score > 0.5 are considered mostly in the same fold, while TM-score < 0.5 are considered mainly not in the same fold [36]. Twelve MYOC variants without experimentally determined structures, including 11 missense variants (p.Q48H, p.D208E, p.C245Y, p.G252R, p.S313F, p.E323K, p.T353I, p.G367R, p.P370L, p.D384H, and p.A488V) and 1 nonsense variant (p.Q368*) were selected for AlphaFold2 modeling based on our previous studies and other reports [14,37,38,39], as well as from the UniProt database (ID: Q99972; https://www.uniprot.org, accessed on 11 December 2021). The potential influence of the variants on the protein structure/function was predicted by Polyphen2 [17]. Multiple sequence alignment was conducted by Clustal Omega (https://www.ebi.ac.uk/Tools/msa/clustalo/, accessed on 11 December 2021) to evaluate the conservation of the amino acid residues of the variants across different species.

### 2.4. AMBER Molecular Dynamic Analysis

The MD was carried out by the Amber 20 platform according to our previous work [40]. Briefly, prior to MD simulation, the protein structures were submitted to the Charmm-Gui website (http://www.charmm-gui.org, accessed on 11 December 2021) to prepare the simulation input files prmtop and prmcrd. The protein structure was protonated, solvated in a rectangular water box with TIP3P water molecules, and neutralized with sodium and chloride counter ions. The periodic boundary conditions were subsequently applied to the system, and the Amber force field ff19SB was used to compute the potential energies from the atomic positions. The generated files prmtop and prmcrd were utilized as the starting structure to perform the MD simulation by means of GPU-accelerated PMEMD and sander modules. The setup system was initially relaxed by a 1000-step minimization, followed by equilibration at constant volume for 80 picoseconds (ρs) and at constant pressure for 20 ρs with restraints. After a further 5 nanoseconds (ns) equilibration at constant pressure without restraints, a 500 ns production simulation was carried out, and the atom coordinates were collected as trajectories every 10,000 steps with step size of 0.002 ρs. The MD simulation with 5 ns equilibration and 500 ns production were carried at NPT ensemble using the Berendsen barostat, while NVT equilibration with 80 ρs were performed at NVT ensemble using the Langevin Thermostat. The cutoff distance was 8 Å. The size simulation box varied from 71 × 71 × 71 to 80 × 80 × 80 (nm^3^) depending on the atoms in the system.

The RMSD was used to measure the average changes in the displacement of the atoms for the backbone. The lower the RMSD, the greater the stability of the protein [41]. The root-mean-square fluctuation (RMSF) of the systems relative to each amino acid residue of the complexes was evaluated to compare the fluctuations of the residues. Besides the C- and N-terminal RMSFs being expected to fluctuate maximally, the fluctuations were observed around the loop regions with additional conformational flexibilities.

For the cross-correlation analysis, the dynamic correlation between residues can be obtained from the trajectories after production simulation by use of an R package Bio3D. The NetCDF-formatted trajectory was sampled and converted into the dcd format file. The output dcd file was input into Bio3D and read along with the pdb-formatted simulation initial structure. The frames in the trajectory were superposed, and a function of dynamic cross-correlation matrix was utilized to calculate Cij, a covariance between the fluctuations of two atoms. The resulting Cij was used to plot the matrix.

For the normal mode analysis, ProDy Python was used to integrate the dcd format trajectory file and the pdb structure and output the nmd format file. The resulting nmd file was loaded into VMD by a module of normal mode wizard. A trajectory with initial 5000 frames from 1 to 5000 was used for the normal mode analysis. The selected Cα atoms were presented by tube representation, and three active modes were used for the analysis. The direction of the arrows denoted the motion direction of each residue, while the relative length of the arrows represented the motion amplitude of each residue.

### 2.5. Schrödinger Molecular Docking Analysis

The molecular docking ability of the AlphaFold2-predicted protein structures was evaluated by the Schrödinger Maestro Suite 2021-1 (Schrödinger LLC, New York, NY, USA) [30]. The structures of MYOC proteins were input into the program, and final optimization and minimizations were executed according to Protein Preparation Wizard. The process of protein preparation utilized the options, including assigning the bond orders, adding the hydrogen atoms, treating the formal charges, and abstracting the water molecules. Side chains and loops with missing atoms were also optimized by the protein preparation protocol. The prepared protein structure was used to generate the glide scoring grids for subsequent docking calculations with standard Glide precision [42].

The structures of apigenin (4′,5,7-trihydroxyflavone; PubChem CID: 5280443) and Gw5074 (3-(3,5-dibromo-4-hydroxybenzylidine-5-iodo-1,3-dihydro-indol-2-one); PubChem CID: 5924208), which have been shown as experimentally binding to the MYOC protein [43], were obtained from the PubChem database (https://pubchem.ncbi.nlm.nih.gov/, accessed on 11 December 2021). The ligands were prepared prior to docking using the LigPrep application. LigPrep was used to expand the protonation and tautomeric states at 7.0 ± 2.0 pH. The energy was minimized using the OPLS force field, and the conversion of structures from 2-dimensional to 3-dimensional was performed. The ligand structures prepared by LigPrep were then used for docking.

One of the most crucial aspects in docking is the suitable active site recognition for the ligand molecule binding [44]. The prepared protein structure was used to generate the glide grids for subsequent docking calculations. The receptor grid was generated from the Receptor Grid Generation panel. This panel allows for defining the receptor structure by excluding any co-crystallized ligand that may be present and determining the position and size of the active site. Default parameters were used, and no constraints were included during the grid generation [42].

SiteMap [45] was used to detect the binding sites in the experimentally determined MYOC wild-type 4WXQ structure. A site score of 0.80 was adopted to distinguish between drug-binding and non-drug-binding sites, and 5 binding sites with the 5 highest Site scores were selected for further ligand docking simulations. Ligand docking was performed using the GLIDE module, following the grid-based docking protocol. Molecular docking of apigenin and Gw5074 was performed with a flexible docking parameter on different experimentally determined and AlphaFold2-modeled MYOC protein structures according to the 5 top binding sites identified in the 4WXQ structure. The prepared glide grids were selected for the molecular docking analysis, and each ligand was docked to the MYOC protein individually and generated a best binding pose ligand–protein complex with the minimum docking score and glide energy. Default parameters were used, and no constraints were applied during the docking process.

## 3. Results

### 3.1. Variant Protein Structure Modeling Potential of AlphaFold2

**Figure 1:**
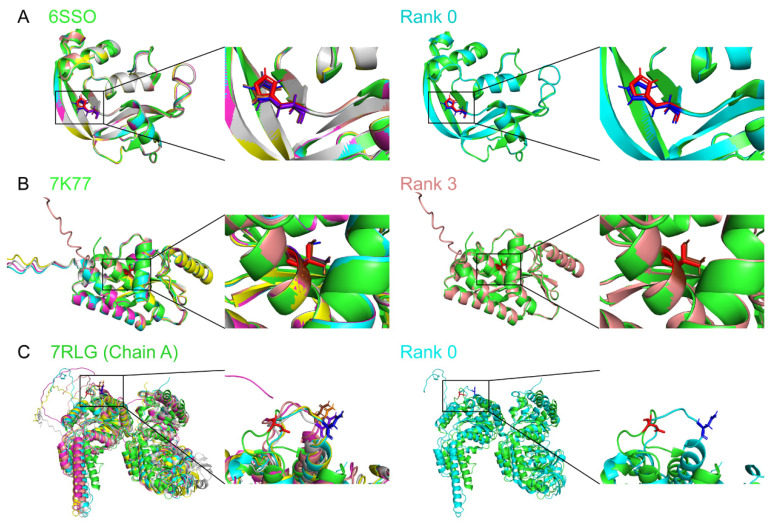
Structure similarity analysis on the AlphaFold2-predicted variant protein structures to the experimentally determined structures from the protein data bank. The 5 AlphaFold2-predicted protein structures (Rank 0–Rank 4) of variants from the protein data bank, (**A**) 6SSO, (**B**) 7K77, and (**C**) 7RLG chain A, were aligned with the corresponding experimentally determined protein structures from the protein data bank (PDB). The alignment of the AlphaFold2-predicted variant protein structures with the highest template modeling score is shown on the right. Green: the PDB structure; Cyan: the AlphaFold2 modeled Rank 0 structure; Magentas: the AlphaFold2 modeled Rank 1 structure; Yellow: the AlphaFold2 modeled Rank 2 structure; Salmon: the AlphaFold2 modeled Rank 3 structure; Light gray: the AlphaFold2 modeled Rank 0 structure; Red: the variant amino acid side chain of the PDB structure; Blue: the variant amino acid side chain of the AlphaFold2 modeled Rank 0 structure; Orange: the variant amino acid side chain of the AlphaFold2 modeled Rank 1 structure; Dark gray: the variant amino acid side chain of the AlphaFold2 modeled Rank 2 structure; Brown: the variant amino acid side chain of the AlphaFold2 modeled Rank 3 structure; Purple: the variant amino acid side chain of the AlphaFold2 modeled Rank 4 structure.

Before working on MYOC protein, we first intended to confirm the potential of AlphaFold2 modeling the variant protein structures. We randomly selected 10 variant protein structures newly released from the PDB after the setup of our AlphaFold2 platform based on the open-source code of AlphaFold2, and their amino acid sequences in the PDB were inputted into our AlphaFold2 platform. Our AlphaFold2 platform would generate 5 modeled structures according to the top 5 pLDDT (per-residue estimate of its confidence; Alphafold confidence scale), and the “Rank 0” structures were presented with the highest pLDDT. All Rank 0 structures of the selected variant proteins achieved a pLDDT higher than 80 (ranged from 81.32 (7RLG chain A) to 98.28 (7JZ7); Supplementary Table S1). However, we found that the modeled variant protein structures with the highest pLDDT did not always show the highest similarity (TM score) as compared to the structures downloaded from the PDB. All modeled variant protein structures achieved the highest TM-score > 0.5 (ranged from 0.768 (7RLG chain A) to 0.998 (7LCL); Supplementary Table S2), indicating that the overall structures of the variant proteins modeled by AlphaFold2 highly resembled the experimentally determined structures from PDB. For the modeled variant structures with high TM-scores, the locations and positions of the variant amino acids in the modeled structures were nearly the same as that of the experimentally determined structures from PDB, but the geometries of the side chains of the amino acids were slightly tilted as compared to that of the PDB structures (Figure 1A (6SSO), Figure 1B (7K77) and Supplementary Figure S1). In contrast, the locations of the variant amino acids and the geometries of the side chains of the amino acids in the modeled structures could be very different from that of the experimentally determined structures from PDB for the modeled structures with a low TM-score (7RLG; Figure 1C). Collectively, we found that most of the AlphaFold2-modeled variant protein structures highly resembled the corresponding structures from PDB.

### 3.2. Myocilin Variant Protein Structure Modeling by AlphaFold2

**Figure 2:**
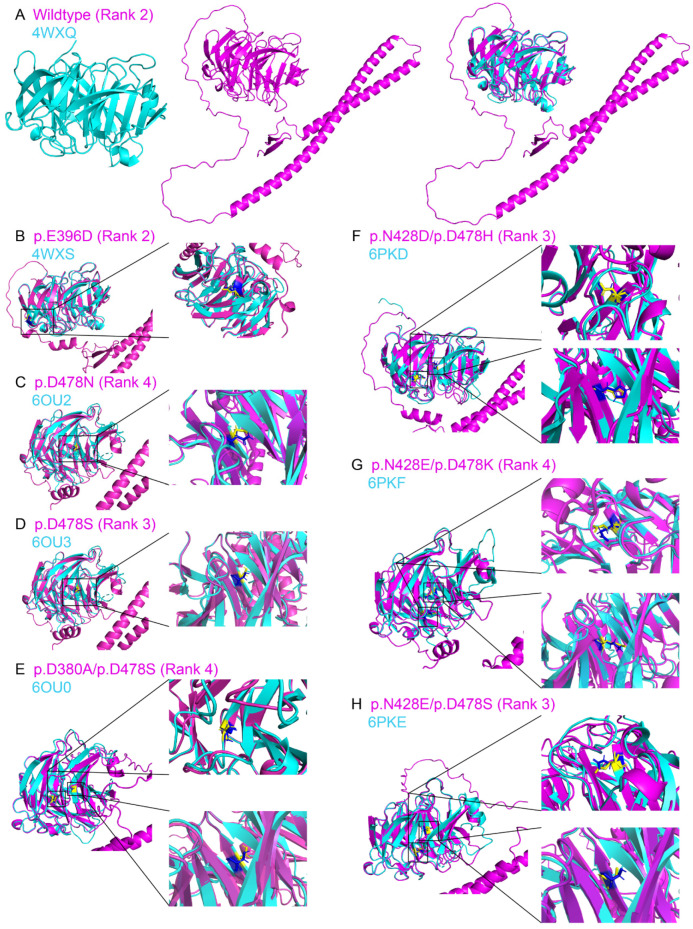
Structure similarity analysis on the AlphaFold2-predicted myocilin wildtype and variant protein structures to the experimentally determined structures. The AlphaFold2-predicted myocilin (**A**) wildtype and variant protein structures ((**B**) p.E396D; (**C**) p.D478N; (**D**) p.D478S; (**E**) p.D380A/p.D478S; (**F**) p.N428D/p.D478H; (**G**) p.N428E/p.D478K; (**H**) p.N428E/p.D478S) with highest template modeling score (red) were aligned with the corresponding experimentally determined protein structures from the protein data bank (green). Yellow: the side chain of the amino acid residue from the experimentally determined protein structure. Blue: the side chain of the amino acid residue from the AlphaFold2-predicted protein structure.

The AlphaFold2-modeled wild-type MYOC protein structure (without signal peptide) achieved the highest pLDDT of 82.08 (Supplementary Table S3) and the highest TM-score of 0.980 (Rank 2) as compared to the experimentally determined structure from the PDB (4WXQ; Figure 2A and Supplementary Table S4), indicating that the AlphaFold2-modeled wildtype MYOC protein structure highly resembles the experimentally determined wildtype MYOC structure from PDB with high confidence.

There were 7 experimentally determined MYOC variant protein structures in the PDB. The AlphaFold2-modeled MYOC variant protein structures achieved the highest pLDDT from 81.90 (6OU2; p.D478N) to 82.99 (6OU0; p.D380A/p.D478S) (Supplementary Table S3) and the highest TM-scores from 0.913 (6PKF; p.N428E/p.D478K) to 0.984 (4WXS; p.E396D) (Figure 2B–H and Supplementary Table S4), indicating that the AlphaFold2-modeled MYOC variant protein structures highly resemble the corresponding experimentally determined MYOC variant structures in PDB.

**Figure 3:**
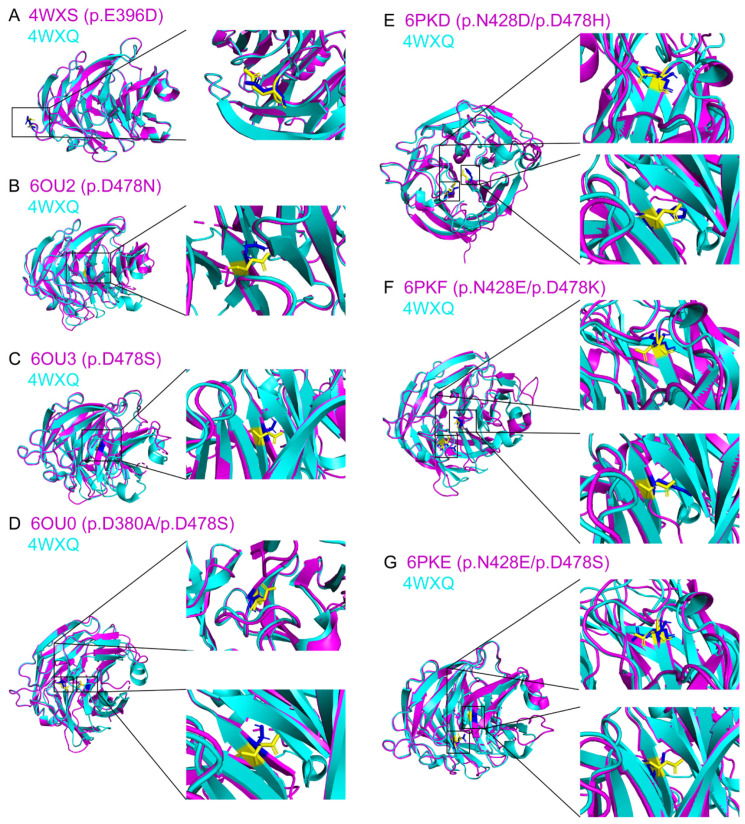
Structure similarity analysis of the experimentally determined myocilin wildtype and variant protein structures. The experimentally determined myocilin variant protein structures ((**A**) p.E396D; (**B**) p.D478N; (**C**) p.D478S; (**D**) p.D380A/p.D478S; (**E**) p.N428D/p.D478H; (**F**) p.N428E/p.D478K; (**G**) p.N428E/p.D478S) (red) were aligned with the experimentally determined myocilin wildtype protein structure (4WXQ) from the protein data bank (green). Yellow: the side chain of the amino acid residue from the wildtype protein structure. Blue: the side chain of the amino acid residue from the variant protein structures.

To evaluate whether the changes in amino acid residues would influence the AlphaFold2-modeled overall protein structures, we first compared the structural similarities of the experimentally determined MYOC variant structures to the experimentally determined wildtype MYOC structure (4WXQ) in PDB. All variants were predicted to be possibly damaging or probably damaging by Polyphen2 (Table 1), and multiple sequence alignment analysis confirmed that the amino acid residues of the variants were mostly conserved across different species, except p.E396D (Supplementary Figure S2). The experimentally determined protein structure of the variant p.E396D (4WXS), determined in the same experiment of 4WXQ, showed almost the same structure as the wildtype MYOC structure with the TM-score of 0.998 and local Distance Difference Test (lDDT) of 0.958 (Supplementary Table S5 and Figure 3). For the single amino acid residue substitution, the experimentally determined protein structures of the variants p.D478N (6OU2) and p.D478S (6OU3) showed high similarities to the experimentally determined wild-type structure with the TM-scores of 0.906 and 0.892 and lDDT of 0.779 and 0.758, respectively. For the double amino acid residue substitution, the experimentally determined MYOC variant protein structures also showed high similarities to the experimentally determined wild-type structure with the TM-scores from 0.891 (6OU0; p.D380A/p.D478S) to 0.922 (6PKD; p.N428D/p.D478H) and lDDT from 0.754 (6OU0) to 0.779 (6PKE; p.N428E/p.D478S). Yet, differences were observed in the electron density maps of the experimentally determined variant protein structures as compared to that of the experimentally determined wild-type 4WXQ structure, except 4WXS (Supplementary Figure S3). These indicated that amino acid residue substitution did not vastly affect overall MYOC protein folding based on the comparisons of the experimentally determined structures.

**Figure 4:**
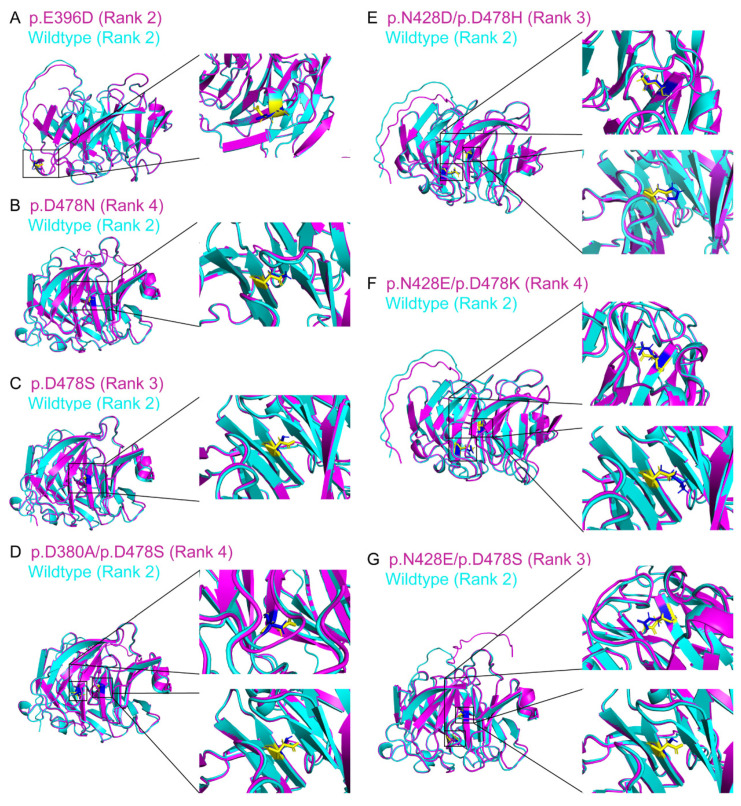
Structure similarity analysis of the AlphaFold2-predicted myocilin wildtype and variant C-terminus protein structures with the protein data bank identities. The AlphaFold2-predicted C-terminus protein structures of myocilin variants ((**A**) p.E396D; (**B**) p.D478N; (**C**) p.D478S; (**D**) p.D380A/p.D478S; (**E**) p.N428D/p.D478H; (**F**) p.N428E/p.D478K; (**G**) p.N428E/p.D478S) with the protein data bank identities (red) were aligned with the AlphaFold2-predicted myocilin wildtype protein structure (Rank 2) (green). Yellow: the side chain of the amino acid residue from the wild-type protein structure. Blue: the side chain of the amino acid residue from the variant protein structures.

For the AlphaFold2-modeled MYOC variant structures, we found that the AlphaFold2-modeled MYOC variant protein structures (residue 33–504; with the highest TM-scores to the experimentally determined structures) showed lower similarity and aligned poorly to the AlphaFold2-modeled wild-type MYOC structure (Rank 2; residue 33–504) with the TM-scores from 0.598 (p.D478N Rank 4) to 0.782 (p.E396D Rank 2) (Supplementary Table S6 and Supplementary Figure S4) as compared to the alignment of the experimentally determined structures from PDB, which could be reflected by different pLDDT in different regions of the AlphaFold2-modeled MYOC protein structures (N-terminal coiled-coil domain: 65.68–78.84; intermediate linker region: 30.11–51.10; C-terminal olfactomedin-like domain: 92.07–96.43) (Supplementary Table S3). With the removal of the modeled N-terminus structure (residue 33–226) based on the calpain II (CAPN2) cleavage, the AlphaFold2-modeled C-terminus MYOC variant protein structures (residue 227–504) showed relatively higher similarities to the AlphaFold2-modeled C-terminus wild-type MYOC structure (Rank 2) with the TM-scores from 0.944 (p.N428E/p.D478S) to 0.987 (p.E396D) and lDDT from 0.929 (p.N428E/p.D478K) to 0.978 (p.E396D) (Table 1 and Figure 4), indicating that the comparisons based on the AlphaFold2-modeled structures are close and consistent with that of the experimentally determined structures.

**Figure 5:**
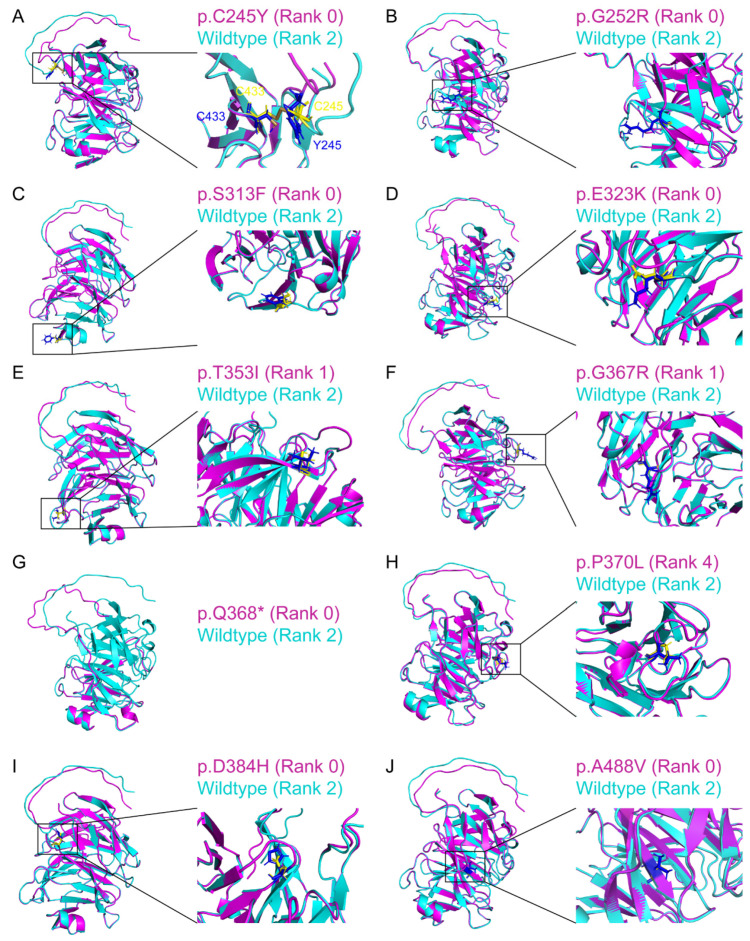
Structure similarity analysis of the AlphaFold2-predicted C-terminus structures of myocilin wildtype and variant proteins without experimentally determined structures. The AlphaFold2-predicted C-terminus protein structures of myocilin variants ((**A**) p.C245Y; (**B**) p.G252R; (**C**) p.S313F; (**D**) p.E323K; (**E**) p.T353I; (**F**) p.G367R; (**G**) p.Q368*; (**H**) p.P370L; (**I**) p.D384H; (**J**) p.A488V) without experimentally determined structures (red) were aligned with the AlphaFold2-predicted myocilin wildtype protein structure (Rank 2) (green). Yellow: the side chain of the amino acid residue from the wildtype protein structure. Blue: the side chain of the amino acid residue from the variant protein structures. Brown: disulfide bond.

Apart from the variants with the experimentally determined protein structures, we further selected another 12 MYOC variants without the experimentally determined structures for AlphaFold2 modeling, including 11 missense variants and 1 nonsense variant (p.Q368*). Among the 11 missense variants, 9 variants located within the olfactomedin-like domain were predicted to be possibly damaging or probably damaging by Polyphen2, and 1 variant (p.Q48H) was predicted to be benign (Table 1). These 11 AlphaFold2-modeled MYOC variant protein structures achieved the highest pLDDT from 79.65 (p.Q368*) to 82.97 (p.G252R) (Supplementary Table S3), which were close to the pLDDT for the modeling of MYOC wild-type and variant protein structures with the experimentally determined structures from PDB. Structural similarity analysis demonstrated that, compared to the AlphaFold2-modeled wild-type MYOC structure (Rank 2; with highest TM-score to the experimentally determined structure), the AlphaFold2-modeled MYOC variant protein structures (with highest pLDDT) achieved the TM-scores from 0.301 (p.Q368*) to 0.769 (p.C245Y) (Supplementary Table S7 and Supplementary Figure S5). Consistently, with the removal of the N-terminus MYOC protein (residue 33–226), the AlphaFold2-modeled C-terminus MYOC variant protein structures (residue 227–504) demonstrated high similarities to the AlphaFold2-modeled C-terminus wild-type MYOC structure (Rank 2) with the TM-scores from 0.949 (p.G367R) to 0.988 (p.S313F), except p.Q368* (TM-score: 0.452) (Table 1 and Figure 5). From the current analyses, our results suggested that MYOC protein with single amino acid substitution likely shows a similar fold as the wildtype MYOC protein.

We further compared the AlphaFold2-modeled MYOC variant protein structures to the MYOC wild-type structure (4WXQ) with amino acid substitutions generated by PyMOL. The structure alignment analysis demonstrated that, even though the backbones of the MYOC olfactomedin-like domain are similar, the orientations and geometries of the amino acid side chains could be different (Supplementary Figures S6 and S7). Our results suggested that AlphaFold2-modeled variant protein structures showed different amino acid geometry and orientation as compared to that with amino acid substitution based on an existing protein backbone.

### 3.3. Molecular Dynamics of AlphaFold2-Predicted Myocilin Protein Structures

**Figure 6:**
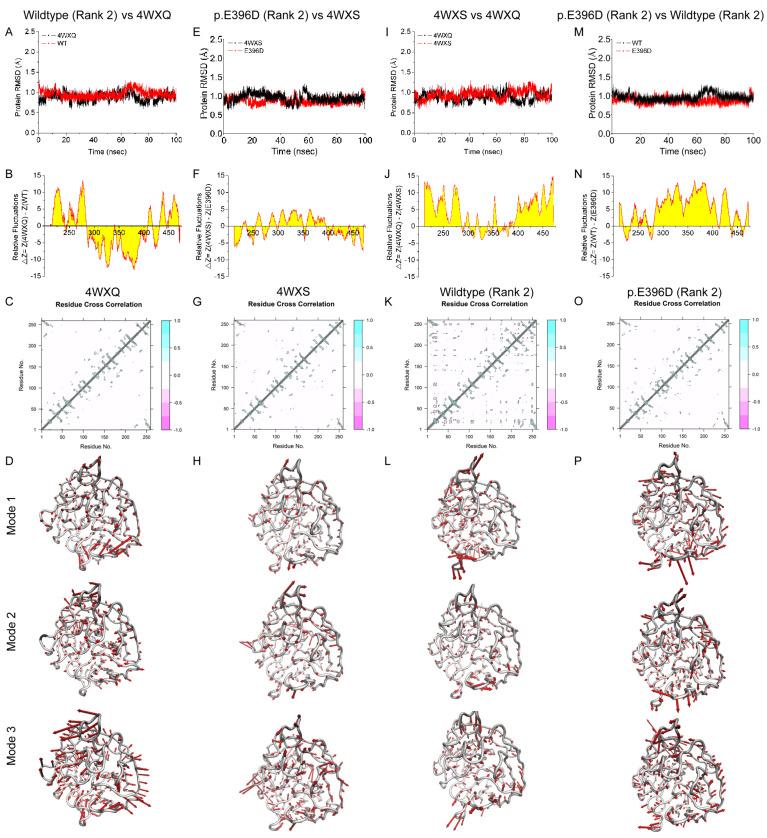
Molecular dynamics analysis on the experimentally determined and AlphaFold2-predicted myocilin wildtype and variant protein structures. (**A**,**E**,**I**,**M**) Root-mean-square deviation (RMSD) analysis. (**B**,**F**,**J**,**N**) Root-mean-square fluctuation (RMSF) analysis. (**C**,**G**,**K**,**O**) residue cross-correlation analysis. (**D**,**H**,**L**,**P**) normal mode analysis. (**A**,**B**) Comparison of the olfactomedin-like domain of the AlphaFold2-predicted wildtype myocilin protein structure (Rank 2) to that of the experimentally determined wild-type myocilin protein structure (4WXQ). (**E**,**F**) Comparison of the olfactomedin-like domain of the AlphaFold2-predicted myocilin p.E396D variant protein structure (Rank 2) to that of the experimentally determined myocilin p.E396D variant protein structure (4WXS). (**I**,**J**) Comparison of the olfactomedin-like domain of the experimentally determined myocilin p.E396D variant protein structure (4WXS) to that of the experimentally determined wild-type myocilin protein structure (4WXQ). (**M**,**N**) Comparison of the olfactomedin-like domain of the AlphaFold2-predicted myocilin p.E396D variant protein structure (Rank 2) to that of the AlphaFold2-predicted wild-type myocilin protein structure (Rank 2). (**C**,**D**) The experimentally determined wildtype myocilin protein structure (4WXQ). (**G**,**H**) The experimentally determined myocilin p.E396D variant protein structure (4WXS). (**K**,**L**) The AlphaFold2-predicted wildtype myocilin protein structure (Rank 2). (**O**,**P**) The AlphaFold2-predicted myocilin p.E396D variant protein structure (Rank 2).

To characterize the molecular properties of the AlphaFold2-modeled MYOC protein structures, the MD simulations of the AlphaFold2-modeled protein structures were simulated by AMBER and compared to the experimentally determined protein structures from PDB. The olfactomedin-like domain of the AlphaFold2-modeled wild-type MYOC structure (Rank 2; with the highest TM-score to the experimentally determined structure) exhibited similar RMSD as compared to that of the experimentally determined wildtype MYOC structure (4WXQ) (Figure 6A) and the AlphaFold2-modeled MYOC wildtype Rank 0 structure (Supplementary Figure S8). Yet, the AlphaFold2-modeled wildtype MYOC structure (Rank 2) showed fluctuating RMSF (Figure 6B), more residue cross-correlation (Figure 6C,K), and different normal modes (Figure 6D,L) as compared to that of the experimentally determined wild-type MYOC structure (4WXQ). In contrast, the olfactomedin-like domain of the AlphaFold2-modeled MYOC p.E396D variant structure (Rank 2) demonstrated similar RMSD (Figure 6E), RMSF (Figure 6F), and residue cross-correlation (Figure 6G,O) but different normal modes (Figure 6H,P) as compared to that of the experimentally determined MYOC p.E396D variant structure (4WXS). For other MYOC variants (p.D380A/p.D478S, p.N428E/p.D478K, and p.N428E/p.D478S), the olfactomedin-like domain of the AlphaFold2-modeled structures showed lower RMSD as compared to their respective experimentally determined variant structures (6OU0, 6PKF, and 6PKE) (Supplementary Figure S9). Higher RMSF was found in the comparison of the AlphaFold2-modeled p.D380A/p.D478S structure with the 6OU0 structure and the AlphaFold2-modeled p.N428E/p.D478S structure with the 6PKE structure, while lower RMSF was found in the comparison of the AlphaFold2-modeled p.N428E/p.D478K structure with the 6PKF structure (Supplementary Figure S10). The AlphaFold2-modeled p.D380A/p.D478S structure showed less residue cross-correlation as compared to the 6OU0 structure, while the AlphaFold2-modeled p.N428E/p.D478K and p.N428E/p.D478S structures showed similar residue cross-correlation as compared to their respective experimentally determined variant structures (6PKF and 6PKE) (Supplementary Figure S11). Different normal modes were also observed.

For the comparison of the MYOC wild-type and variant structures, the olfactomedin-like domain of the experimentally determined MYOC p.E396D variant structure (4WXS) showed similar RMSD (Figure 6I) and residue cross-correlation (Figure 6C,G), but higher RMSF (Figure 6J) and different normal modes (Figure 6D,H) as compared to that of the experimentally determined wild-type MYOC structure (4WXQ). For other MYOC variants, the experimentally determined variant structures (6OU0 (p.D380A/p.D478S), 6PKF (p.N428E/p.D478K), and 6PKE (p.N428E/p.D478S)) showed higher RMSD as compared to the experimentally determined wildtype MYOC structure (4WXQ) (Supplementary Figure S9). The 6PKF structure showed higher RMSF than the 4WXQ structure, while fluctuating RMSF was found in the comparison of 6OU0 and 6PKE with 4WXQ structures (Supplementary Figure S10). All 6OU0, 6PKF, and 6PKE structures showed more residue cross-correlation as compared to the experimentally determined wildtype MYOC structure (4WXQ) (Supplementary Figure S11). Different normal modes were also observed.

For the AlphaFold2-modeled structures, the olfactomedin-like domain of the AlphaFold2-modeled MYOC p.E396D variant structure exhibited similar RMSD (Figure 6M), but higher RMSF (Figure 6N), less residue cross-correlation (Figure 6K,O), and different normal modes (Figure 6L,P) as compared to that the AlphaFold2-modeled wild-type MYOC structure. For other MYOC variants (p.D380A/p.D478S, p.N428E/p.D478K, and p.N428E/p.D478S), the olfactomedin-like domain of the AlphaFold2-modeled structures showed similar RMSD as compared to the AlphaFold2-modeled wild-type MYOC structure (Supplementary Figure S9). The AlphaFold2-modeled p.D380A/p.D478S and p.N428E/p.D478S structures showed higher RMSF than the AlphaFold2-modeled wildtype MYOC structure, while fluctuating RMSF was found in the comparison of the AlphaFold2-modeled p.N428E/p.D478K structure with the AlphaFold2-modeled wild-type MYOC structure (Supplementary Figure S10). The AlphaFold2-modeled p.D380A/p.D478S and p.N428E/p.D478K structures showed less residue cross-correlation while the AlphaFold2-modeled p.N428E/p.D478S structure showed more residue cross-correlation as compared to the AlphaFold2-modeled wild-type MYOC structure (Supplementary Figure S11). Different normal modes were also observed.

Our results suggested that the AlphaFold2-modeled MYOC structures exhibited different MD simulation properties as compared to the experimentally determined structures, and different MD simulation properties were also found between the MYOC wild-type and variant structures.

### 3.4. Molecular Docking of AlphaFold2-Predicted Myocilin Protein Structures

**Figure 7:**
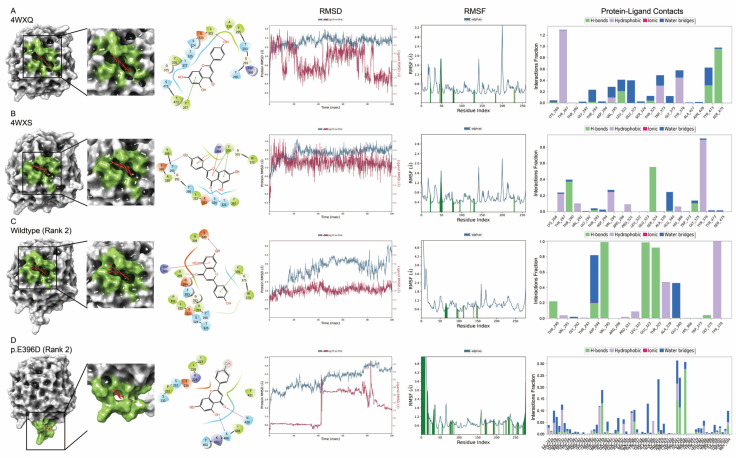
Molecular docking analysis of apigenin on the experimentally determined and AlphaFold2-predicted myocilin wildtype and variant protein structures. Molecular docking analysis of apigenin (red) on the experimentally determined myocilin (**A**) wildtype (4WXQ) and (**B**) variant (4WXS; p.E396D) protein structures and the AlphaFold2-predicted C-terminus protein structures of myocilin (**C**) wild-type (Rank 2) and (**D**) p.E396D variant (Rank 2). The surface structural representation, binding site (green), protein–ligand root-mean-square deviation (RMSD), protein root-mean-square fluctuation (RMSF), and protein–ligand contacts are shown.

**Figure 8:**
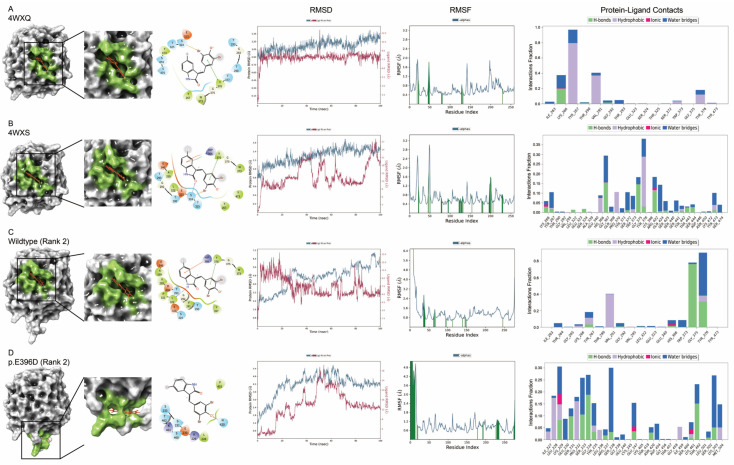
Molecular docking analysis of Gw5074 on the experimentally determined and AlphaFold2-predicted myocilin wildtype and variant protein structures. Molecular docking analysis of Gw5074 (orange) on the experimentally determined myocilin (**A**) wildtype (4WXQ) and (**B**) variant (4WXS; p.E396D) protein structures and the AlphaFold2-predicted C-terminus protein structures of myocilin (**C**) wild-type (Rank 2) and (**D**) p.E396D variant (Rank 2). The surface structural representation, binding site (green), protein–ligand root-mean-square deviation (RMSD), protein root-mean-square fluctuation (RMSF), and protein–ligand contacts are shown.

To further elucidate the molecular properties of the AlphaFold2-modeled MYOC protein structures, the molecular docking properties of the AlphaFold2-modeled protein structures were evaluated by the Schrödinger simulation analysis and compared to the experimentally determined protein structures from PDB. Without specific site allocation, apigenin and Gw5074 demonstrated the docking score of −7.04 (interacting with Y267 and L322) and −4.88 (interacting with S324 and Y376), respectively, on the experimentally determined wildtype MYOC structure (4WXQ) with the highest site scores of 1.07 (Supplementary Table S8). As compared to the experimentally determined wild-type MYOC structure (4WXQ), the structure 4WXS (p.E396D) should have stronger binding of Gw5074 (docking score: −5.38) to the similar site (site score: 0.88) interacting with S324, H366, and Y376 (Figure 7B and Figure 8B). For apigenin, higher ligand RMSD and less protein–ligand contact, with similar RMSF, was found in the 4WXS structure as compared to the wild-type 4WXQ structure (Figure 7A). In contrast, for Gw5074, lower ligand RMSD and more protein–ligand contact were found in the 4WXS structure as compared to the wild-type 4WXQ structure (Figure 8A). Other experimentally determined MYOC variant structures showed different binding sites and protein–ligand molecular dynamics properties for apigenin (Supplementary Figure S12) and Gw5074 (Supplementary Figure S13), implying that the experimentally determined MYOC variant proteins could have different conformations and side chain geometries to the experimentally determined wildtype MYOC structure.

For the AlphaFold2-modeled structures, the binding site (site score: 0.92) of apigenin (docking score of −6.65, interacting with D294, V295, E340E, H366, and Y376) and Gw5074 (docking score of −5.68, interacting with L322, H366, and Y376) on the AlphaFold2-modeled C-terminus MYOC wild-type structure (Rank 2) with different interacting amino acids and protein–ligand molecular dynamics properties as compared to the experimentally determined 4WXQ and 4WXS structures (Figure 7C and Figure 8C and Supplementary Table S9). Additionally, the AlphaFold2-modeled MYOC variant protein structures showed different binding sites of apigenin and Gw5074 to their counterpart experimentally determined structures with different protein–ligand molecular dynamics properties (Figure 7D and Figure 8D and Supplementary Figures S14 and S15), indicating that the conformations and side chain geometries of the AlphaFold2-modeled MYOC wildtype and variant proteins were not exactly the same as the experimentally determined MYOC protein structures.

## 4. Discussion

Results from this study demonstrated that: (1) the variant protein structures modeled by AlphaFold2 mostly resemble the experimentally determined structures with nearly the same locations and positions of the variant amino acids as well as the side chain geometries; (2) the AlphaFold2-modeled MYOC protein structures with missense variants are mostly in the same fold with that of the wild-type protein structure but not for the nonsense variant; (3) the AlphaFold2-modeled MYOC structures exhibit different MD simulation properties from the experimentally determined structures; (4) the AlphaFold2-modeled MYOC structures show different ligand binding sites and protein–ligand MD to the experimentally determined structures. Collectively, this study demonstrated the application of AlphaFold2 on variant protein structure modeling with subsequent molecular simulation analyses.

The complete crystal structure of the full-length human myocilin protein still remains unsolved. With the application of AlphaFold2 modeling, we, for the first time, proposed the full-length structure of human MYOC protein (without the inclusion of the signal peptide in the prediction) (Figure 2) that the AlphaFold2-modeled structures showed high similarity in global fold and details (TM-score > 0.9) as compared to the olfactomedin-like domain of the experimentally determined structures of MYOC wildtype (4WXQ) and variant proteins (Supplementary Table S4). Moreover, the N-terminus portion (33–201 residues) of the AlphaFold2-modeled structure of wild-type MYOC protein was predicted to be composed of one short and one long helices, connecting the olfactomedin-like domain with a loop (residue 202–243) (Figure 2A). Different confident scores (pLDDT) were observed in different regions of the AlphaFold2-modeled MYOC protein structures (Supplementary Table S3) that the structure modeling on the C-terminal olfactomedin-like domain has higher confidence than that on the N-terminal coiled-coil domain.

Concerns have been raised about whether protein structures with missense mutations or splice variants could be modeled by AlphaFold2 [46]. In this study, we found that the olfactomedin-like domain of experimentally determined MYOC variant structures folded mostly in the same way as that of the wild-type structure (Supplementary Table S5) but with different side chain orientation and geometry (Figure 3) as well as differences in the electron density maps (Supplementary Figure S3). A similar scenario was also observed in the comparison of the AlphaFold2-modeled MYOC wildtype and variant protein structures (Table 1 and Figure 4). We also demonstrated that the side chain orientation and geometry of the variants in the AlphaFold2-modeled structures were different from that of the amino acid substitution generated on the experimentally determined structures (Supplementary Figures S6 and S7). The conformational differences between the MYOC wild-type and variant structures could be further observed in the MD analysis that different experimentally determined variant structures showed different conformational fluctuation, flexibility, residue cross-correlation, and patterns of motions as compared to that of the wild-type 4WXQ structure (Figure 6 and Supplementary Figures S9–11). In contrast, different AlphaFold2-predicted MYOC variant structures exhibited similar conformational fluctuation but different structural flexibility, residue cross-correlation, and patterns of motions as compared to the AlphaFold2-predicted wildtype MYOC structure (Figure 6 and Supplementary Figures S9–S11). Moreover, different ligand binding sites and protein–ligand MD were also found between the experimentally determined MYOC wild-type and variant structures and the AlphaFold2-modeled MYOC wild-type and variant structures (Figure 7 and Figure 8 and Supplementary Figures S12–S15). Our results could indicate the conformational differences between the MYOC wildtype and variant proteins as well as between the experimentally determined and AlphaFold2-modeled structures. This could be due to that current protein structure modeling methods, including AlphaFold, focus on predicting the backbone structure of proteins correctly without emphasizing the improvement of the nativeness and all-atom geometry of predicted structures [47]. Furthermore, X-ray crystallography involves crystal formation so that the conformation of the experimentally determined protein structure might not actually reflect the biological conformation [24]. 

AlphaFold has been applied to model the protein structure of gene mutation from a patient with cognitive developmental delay [48]. In this study, we applied AlphaFold2 to model the protein structures of 12 MYOC variants without previous experimentally determined structures (Supplementary Figure S5). All non-synonymous missense variants in the olfactomedin-like domain were predicted as probably or possibly damaging by Polyphen2 (Table 1), but their AlphaFold2-predicted structures all showed high similarity in global fold as compared to the AlphaFold2-predicted MYOC wildtype structure (Figure 5). Yet, different ligand binding sites and protein–ligand MD in the molecular docking analysis could suggest prominent conformational differences in the variant protein structures (Figure 7 and Figure 8, Supplementary Figures S14 and S15, and Supplementary Table S9), although the actual binding site of the ligands on MYOC protein has still not been reported. Further investigations can elucidate the real binding site of the ligands on MYOC protein and evaluate the binding potentials of different ligands on MYOC protein in addition to apigenin (a plant-derived flavonoid promising for the POAG treatment [49]) and Gw5074. Nevertheless, AlphaFold2 modeling together with in silico molecular simulation could be a new strategy to evaluate the deleterious effect or the pathogenicity of the gene variants.

Similar to our study, a recent report on the use of AlphaFold2 modeling in the study of characteristic structural elements, the impact of missense variants, function and ligand binding site predictions, modeling of interactions, and modeling of experimental structural data indicated that AlphaFold2 models can be used across diverse applications equally well compared with experimentally determined structures when the confidence metrics are critically considered [50]. However, AlphaFold2 guidelines stated that “AlphaFold has not been validated for predicting the effect of mutations. In particular, AlphaFold is not expected to produce an unfolded protein structure given a sequence containing a destabilising point mutation.” An earlier study compared the AlphaFold2-modeled structures of known mutants in the ubiquitin-associated domains of human Rad23 protein, breast cancer 1 (BRCA1) C-terminal repeats of BRCA1, and the Myosin VI MyUb domain to their wild-type counterparts and concluded that AlphaFold2 is currently unable to predict when a point mutation causes defective protein folding [51]. Moreover, a study on the use of AlphaFold to predict the impact of single mutations on protein stability and function found very weak or no correlation between AlphaFold output metrics and change in protein stability or fluorescence [52]. Further experiments are still needed to show the pathogenicity of the gene variants and whether the AlphaFold2-modeled variant structures would be experimentally correct [53].

In summary, this study revealed that the folding of the AlphaFold2-modeled MYOC variant protein structures is similar to the experimentally determined structures, but the orientations and geometries of amino acid side chains and the MD and ligand binding properties could be different. Careful comparisons with the experimentally determined structures are needed before the applications of the in silico-modeled variant protein structures.

## Figures and Tables

**Table 1:** Structure similarity analysis of the AlphaFold2-predicted myocilin wildtype and variant C-terminus protein structures.

Variants	PDB ID	Length (Residues)	Polyphen2 HumDiv Score	Polyphen2 Prediction	Rank	TM-Score	lDDT	RMSD
p.E396D	4WXS	277	0.657	Probably damaging	Rank 2	0.987	0.978	0.65
p.D478N	6OU2	277	1.000	Probably damaging	Rank 4	0.981	0.936	0.63
p.D478S	6OU3	277	1.000	Possibly damaging	Rank 3	0.945	0.938	0.96
p.D380A/p.D478S	6OU0	277	1.000/1.000	Probably damaging	Rank 4	0.944	0.934	0.59
p.N428D/p.D478H	6PKD	277	1.000/1.000	Possibly damaging	Rank 3	0.972	0.933	1.1
p.N428E/p.D478K	6PKF	277	1.000/1.000	Probably damaging	Rank 4	0.976	0.929	0.93
p.N428E/p.D478S	6PKE	277	1.000/1.000	Probably damaging	Rank 3	0.944	0.926	0.62
p.C245Y	/	277	1.000	Probably damaging	Rank 0	0.963	0.973	1.21
p.G252R	/	277	1.000	Probably damaging	Rank 0	0.980	0.985	1.03
p.S313F	/	277	0.999	Probably damaging	Rank 0	0.988	0.985	0.67
p.E323K	/	277	1.000	Probably damaging	Rank 0	0.975	0.969	0.94
p.T353I	/	277	0.560	Possibly damaging	Rank 1	0.977	0.971	0.94
p.G367R	/	277	1.000	Probably damaging	Rank 1	0.987	0.980	0.67
p.Q368*	/	141	/	/	Rank 0	0.452	0.307	1.15
p.P370L	/	277	1.000	Probably damaging	Rank 4	0.980	0.938	0.91
p.D384H	/	277	1.000	Probably damaging	Rank 0	0.973	0.973	1.21
p.A488V	/	277	0.999	Probably damaging	Rank 0	0.974	0.975	1.15

The AlphaFold2-predicted myocilin variant structures (residue 227–504) were compared to the AlphaFold2-predicted myocilin wildtype protein structure (Rank 2; with highest template modeling (TM)-score to the experimentally determined structure). lDDT: local Distance Difference Test; Polyphen2: Polymorphism Phenotyping version 2; PDB: Protein data bank; RMSD: root-mean-square deviation.

## Data Availability

The data can be made available upon reasonable request to the corresponding author.

## Supplementary Materials

The following supporting information can be downloaded at: https://www.mdpi.com/article/10.3390/biom14010014/s1, Figure S1: Structure similarity analysis on the AlphaFold2-predicted variant protein structures to the experimentally determined structures from protein data bank; Figure S2: Multiple sequence analysis on myocilin variant sites; Figure S3: Electron density maps of the experimentally determined myocilin wildtype and variant protein structures; Figure S4: Structure similarity analysis of the AlphaFold2-predicted full-length myocilin wildtype and variant protein structures with protein data bank identities; Figure S5: Structure similarity analysis of the AlphaFold2-predicted full-length structures of myocilin wildtype and variant proteins without experimentally determined structures; Figure S6: Structure similarity analysis on the C-terminus of AlphaFold2-predicted myocilin variant protein structures with protein data bank identities to the amino acid substitution on the experimentally determined myocilin wildtype structure; Figure S7: Structure similarity analysis on the C-terminus of AlphaFold2-predicted myocilin variant protein structures without previous experimental determination to the amino acid substitution on the experimentally determined myocilin wildtype structure; Figure S8: Root mean square deviation analysis of molecular dynamics on the experimentally determined and AlphaFold2-predicted wildtype myocilin protein structures; Figure S9: Root mean square deviation analysis of molecular dynamics on the experimentally determined and AlphaFold2-predicted myocilin wildtype and variant protein structures; Figure S10: Root mean square fluctuation analysis of molecular dynamics on the experimentally determined and AlphaFold2-predicted myocilin wildtype and variant protein structures; Figure S11: Residue cross-correlation and normal mode analyses of molecular dynamics on the experimentally determined and AlphaFold2-predicted myocilin variant protein structures; Figure S12: Molecular docking analysis of apigenin on the experimentally determined myocilin variant protein structures; Figure S13: Molecular docking analysis of Gw5074 on the experimentally determined myocilin variant protein structures; Figure S14: Molecular docking analysis of apigenin on the AlphaFold2-predicted myocilin variant protein structures; Figure S15: Molecular docking analysis of Gw5074 on the AlphaFold2-predicted myocilin variant protein structures; Table S1: Confident score of AlphaFold2 prediction on 10 experimentally determined variant protein structures from the protein data bank; Table S2: Structure similarity analysis of AlphaFold2 prediction on 10 experimentally determined variant protein structures from the protein data bank; Table S3: Confident score of AlphaFold2 prediction on myocilin wildtype and variant protein structures; Table S4: Structure similarity analysis of AlphaFold2 prediction on the experimentally determined myocilin wildtype and variant protein structures from the protein data bank; Table S5: Structure similarity analysis of the experimentally determined myocilin wildtype and variant protein structures from the protein data bank; Table S6: Structure similarity analysis of the AlphaFold2-predicted full-length myocilin wildtype and variant protein structures with protein data bank identities; Table S7: Structure similarity analysis of the AlphaFold2-predicted full-length myocilin variant protein structures without experimentally determined structures; Table S8: Molecular docking analysis on the experimentally determined myocilin wildtype and variant protein structures; Table S9: Molecular docking analysis on the AlphaFold2-predicted myocilin wildtype and variant protein structures.

## References

[1] Kapetanakis V.V., Chan M.P., Foster P.J., Cook D.G., Owen C.G., Rudnicka A.R. (2016). Global variations and time trends in the prevalence of primary open angle glaucoma (POAG): A systematic review and meta-analysis. Br. J. Ophthalmol..

[2] Weinreb R.N., Aung T., Medeiros F.A. (2014). The pathophysiology and treatment of glaucoma: A review. JAMA.

[3] Kubota R., Noda S., Wang Y., Minoshima S., Asakawa S., Kudoh J., Mashima Y., Oguchi Y., Shimizu N. (1997). A novel myosin-like protein (myocilin) expressed in the connecting cilium of the photoreceptor: Molecular cloning, tissue expression, and chromosomal mapping. Genomics.

[4] Stone E.M., Fingert J.H., Alward W.L., Nguyen T.D., Polansky J.R., Sunden S.L., Nishimura D., Clark A.F., Nystuen A., Nichols B.E. (1997). Identification of a gene that causes primary open angle glaucoma. Science.

[5] Gupta V., Somarajan B.I., Gupta S., Walia G.K., Singh A., Sofi R., Chaudhary R.S., Sharma A. (2021). The mutational spectrum of Myocilin gene among familial versus sporadic cases of Juvenile onset open angle glaucoma. Eye.

[6] Ortego J., Escribano J., Coca-Prados M. (1997). Cloning and characterization of subtracted cDNAs from a human ciliary body library encoding TIGR, a protein involved in juvenile open angle glaucoma with homology to myosin and olfactomedin. FEBS Lett..

[7] Sharma R., Grover A. (2021). Myocilin-Associated Glaucoma: A Historical Perspective and Recent Research Progress. Mol. Vis..

[8] Sánchez-Sánchez F., Martínez-Redondo F., Aroca-Aguilar J.D., Coca-Prados M., Escribano J. (2007). Characterization of the intracellular proteolytic cleavage of myocilin and identification of calpain II as a myocilin-processing protease. J. Biol. Chem..

[9] Aroca-Aguilar J.D., Sánchez-Sánchez F., Ghosh S., Coca-Prados M., Escribano J. (2005). Myocilin mutations causing glaucoma inhibit the intracellular endoproteolytic cleavage of myocilin between amino acids Arg226 and Ile227. J. Biol. Chem..

[10] Hill S.E., Nguyen E., Donegan R.K., Patterson-Orazem A.C., Hazel A., Gumbart J.C., Lieberman R.L. (2017). Structure and Misfolding of the Flexible Tripartite Coiled-Coil Domain of Glaucoma-Associated Myocilin. Structure.

[11] Donegan R.K., Hill S.E., Freeman D.M., Nguyen E., Orwig S.D., Turnage K.C., Lieberman R.L. (2015). Structural basis for misfolding in myocilin-associated glaucoma. Hum. Mol. Genet..

[12] Hewitt A.W., Mackey D.A., Craig J.E. (2008). Myocilin allele-specific glaucoma phenotype database. Hum. Mutat..

[13] Gong G., Kosoko-Lasaki O., Haynatzki G.R., Wilson M.R. (2004). Genetic dissection of myocilin glaucoma. Hum. Mol. Genet..

[14] Liuska P.J., Lemmelä S., Havulinna A.S., Kaarniranta K., Uusitalo H., Laivuori H., Kiiskinen T., Daly M.J., Palotie A., Turunen J.A. (2021). Association of the MYOC p.(Gln368Ter) Variant with Glaucoma in a Finnish Population. JAMA Ophthalmol..

[15] Scelsi H.F., Barlow B.M., Saccuzzo E.G., Lieberman R.L. (2021). Common and rare myocilin variants: Predicting glaucoma pathogenicity based on genetics, clinical, and laboratory misfolding data. Hum. Mutat..

[16] Pang C.P., Leung Y.F., Fan B., Baum L., Tong W.C., Lee W.S., Liu Y., Lam D.S. (2002). TIGR/MYOC gene sequence alterations in individuals with and without primary open-angle glaucoma. Investig. Ophthalmol. Vis. Sci..

[17] Ramensky V., Bork P., Sunyaev S. (2002). Human non-synonymous SNPs: Server and survey. Nucleic Acids Res..

[18] Ng P.C., Henikoff S. (2001). Predicting deleterious amino acid substitutions. Genome Res..

[19] Schwarz J.M., Cooper D.N., Schuelke M., Seelow D. (2014). MutationTaster2: Mutation prediction for the deep-sequencing age. Nat. Methods.

[20] Rentzsch P., Witten D., Cooper G.M., Shendure J., Kircher M. (2019). CADD: Predicting the deleteriousness of variants throughout the human genome. Nucleic Acids Res..

[21] Bijak V., Szczygiel M., Lenkiewicz J., Gucwa M., Cooper D.R., Murzyn K., Minor W. (2023). The current role and evolution of X-ray crystallography in drug discovery and development. Expert Opin. Drug Discov..

[22] Ravera E., Gigli L., Fiorucci L., Luchinat C., Parigi G. (2022). The evolution of paramagnetic NMR as a tool in structural biology. Phys. Chem. Chem. Phys..

[23] Cheng Y. (2015). Single-Particle Cryo-EM at Crystallographic Resolution. Cell.

[24] Pearce R., Zhang Y. (2021). Toward the solution of the protein structure prediction problem. J. Biol. Chem..

[25] Jumper J., Evans R., Pritzel A., Green T., Figurnov M., Ronneberger O., Tunyasuvunakool K., Bates R., Žídek A., Potapenko A. (2021). Highly accurate protein structure prediction with AlphaFold. Nature.

[26] Baek M., DiMaio F., Anishchenko I., Dauparas J., Ovchinnikov S., Lee G.R., Wang J., Cong Q., Kinch L.N., Schaeffer R.D. (2021). Accurate prediction of protein structures and interactions using a three-track neural network. Science.

[27] Anfinsen C.B. (1973). Principles that govern the folding of protein chains. Science.

[28] Berman H.M., Westbrook J., Feng Z., Gilliland G., Bhat T.N., Weissig H., Shindyalov I.N., Bourne P.E. (2000). The Protein Data Bank. Nucleic Acids Res..

[29] Case D.A., Cheatham T.E., Darden T., Gohlke H., Luo R., Merz K.M., Onufriev A., Simmerling C., Wang B., Woods R.J. (2005). The Amber biomolecular simulation programs. J. Comput. Chem..

[30] Friesner R.A., Banks J.L., Murphy R.B., Halgren T.A., Klicic J.J., Mainz D.T., Repasky M.P., Knoll E.H., Shelley M., Perry J.K. (2004). Glide: A new approach for rapid, accurate docking and scoring. 1. Method and assessment of docking accuracy. J. Med. Chem..

[31] Hill S.E., Kwon M.S., Martin M.D., Suntharalingam A., Hazel A., Dickey C.A., Gumbart J.C., Lieberman R.L. (2019). Stable calcium-free myocilin olfactomedin domain variants reveal challenges in differentiating between benign and glaucoma-causing mutations. J. Biol. Chem..

[32] Hill S.E., Cho H., Raut P., Lieberman R.L. (2019). Calcium-ligand variants of the myocilin olfactomedin propeller selected from invertebrate phyla reveal cross-talk with N-terminal blade and surface helices. Acta Crystallogr. D Struct. Biol..

[33] Zhang Y., Skolnic J. (2004). Scoring function for automated assessment of protein structure template quality. Proteins.

[34] Mariani V., Biasini M., Barbato A., Schwede T. (2013). lDDT: A local superposition-free score for comparing protein structures and models using distance difference tests. Bioinformatics.

[35] Zemla A. (2003). LGA: A method for finding 3D similarities in protein structures. Nucleic Acids Res..

[36] Xu J., Zhang Y. (2010). How significant is a protein structure similarity with TM-score = 0.5?. Bioinformatics.

[37] Huang C., Xie L., Wu Z., Cao Y., Zheng Y., Pang C.P., Zhang M. (2018). Detection of mutations in MYOC, OPTN, NTF4, WDR36 and CYP1B1 in Chinese juvenile onset open-angle glaucoma using exome sequencing. Sci. Rep..

[38] Fan B.J., Leung D.Y., Wang D.Y., Gobeil S., Raymond V., Tam P.O., Lam D.S., Pang C.P. (2006). Novel myocilin mutation in a Chinese family with juvenile-onset open-angle glaucoma. Arch. Ophthalmol..

[39] Jia L.Y., Tam P.O., Chiang S.W., Ding N., Chen L.J., Yam G.H., Pang C.P., Wang N.L. (2009). Multiple gene polymorphisms analysis revealed a different profile of genetic polymorphisms of primary open-angle glaucoma in northern Chinese. Mol. Vis..

[40] Wu X., Wen B., Lin L., Shi W., Li D., Cheng Y., Xu L.Y., Li E.M., Dong G. (2021). New insights into the function of Fascin in actin bundling: A combined theoretical and experimental study. Int. J. Biochem. Cell Biol..

[41] Aier I., Varadwaj P.K., Raj U. (2016). Structural insights into conformational stability of both wild-type and mutant EZH2 receptor. Sci. Rep..

[42] Ahmad S., Khan M.F., Parvez S., Akhtar M., Raisuddin S. (2017). Molecular docking reveals the potential of phthalate esters to inhibit the enzymes of the glucocorticoid biosynthesis pathway. J. Appl. Toxicol..

[43] Orwig S.D., Chi P.V., Du Y., Hill S.E., Cavitt M.A., Suntharalingam A., Turnage K.C., Dickey C.A., France S., Fu H. (2014). Ligands for glaucoma-associated myocilin discovered by a generic binding assay. ACS Chem. Biol..

[44] Yadav R., Imran M., Dhamija P., Chaurasia D.K., Handu S. (2021). Virtual screening, ADMET prediction and dynamics simulation of potential compounds targeting the main protease of SARS-CoV-2. J. Biomol. Struct. Dyn..

[45] Halgren T. (2007). New method for fast and accurate binding-site identification and analysis. Chem. Biol. Drug Des..

[46] Skolnick J., Gao M., Zhou H., Singh S. (2021). AlphaFold 2: Why It Works and Its Implications for Understanding the Relationships of Protein Sequence, Structure, and Function. J. Chem. Inf. Model..

[47] Wu T., Guo Z., Cheng J. (2023). Atomic protein structure refinement using all-atom graph representations and SE(3)-equivariant graph transformer. Bioinformatics.

[48] López-Rivera J.J., Rodríguez-Salazar L., Soto-Ospina A., Estrada-Serrato C., Serrano D., Chaparro-Solano H.M., Londoño O., Rueda P.A., Ardila G., Villegas-Lanau A. (2022). Structural Protein Effects Underpinning Cognitive Developmental Delay of the PURA p.Phe233del Mutation Modelled by Artificial Intelligence and the Hybrid Quantum Mechanics-Molecular Mechanics Framework. Brain Sci..

[49] Zhu J., Chen L., Qi Y., Feng J., Zhu L., Bai Y., Wu H. (2018). Protective effects of Erigeron breviscapus Hand.-Mazz. (EBHM) extract in retinal neurodegeneration models. Mol. Vis..

[50] Akdel M., Pires D.E.V., Pardo E.P., Jänes J., Zalevsky A.O., Mészáros B., Bryant P., Good L.L., Laskowski R.A., Pozzati G. (2022). A structural biology community assessment of AlphaFold2 applications. Nat. Struct. Mol. Biol..

[51] Buel G.R., Walters K.J. (2022). Can AlphaFold2 predict the impact of missense mutations on structure?. Nat. Struct. Mol. Biol..

[52] Pak M.A., Markhieva K.A., Novikova M.S., Petrov D.S., Vorobyev I.S., Maksimova E.S., Kondrashov F.A., Ivankov D.N. (2023). Using AlphaFold to predict the impact of single mutations on protein stability and function. PLoS ONE.

[53] Callaway E. (2022). What’s next for AlphaFold and the AI protein-folding revolution. Nature.

