# Supplementary material for: Evaluation of Myocilin Variant Protein Structures Modeled by AlphaFold2
Source: Biomolecules. 2023 Dec 21;14(1):14. doi: 10.3390/biom14010014 (PMC10813463; doi:10.3390/biom14010014)
Supplement: Supplementary file 1 [file biomolecules-14-00014-s001.zip › biomolecules-2636549-supplementary-conversion.pdf]

Supplementary table 1: Confident score of AlphaFold2 prediction on 10 experimentally determined variant protein structures from the protein data bank.

Organisms	Variant proteins	PDB ID	Size (residues)	Released date	Rank 0	Rank 1	Rank 2	Rank 3	Rank 4
Homo sapiens	EDN mutant L45H	6SSO	135	Oct 6th, 2021	97.81	97.72	97.27	97.18	96.99
Homo sapiens	ubiquitin-conjugating enzyme UBE2S L114E varaiant	7AHF (chain A)	156	Oct 6th, 2021	93.76	93.53	93.48	93.43	93.39
Influenza A virus (A/Luxembourg/43/2009(H1N1))	PA endonuclease mutant I38T in complex with SJ001008025	7K77	197	Sept 22nd, 2021	92.37	91.80	90.99	90.32	89.78
Pseudomonas putida DOT-T1E	TtgR quadruple mutant (C137I I141W M167L F168Y)	7K1A (chain B)	211	Oct 6th, 2021	95.02	94.96	94.53	94.44	93.63
Escherichia coli	Dihydrodipicolinate synthase mutant S48F	7JZ7 (chain A)	292	Oct 6th, 2021	98.28	98.11	97.86	97.74	97.65
Trypanosoma cruzi strain CL Brener	F337L mutation of Trypanosoma cruzi glucokinase in the apo form	7S2N (chain A)	381	Oct 6th, 2021	95.86	95.83	94.05	93.84	93.70
Zoogloea ramigera	biosynthetic thiolase Y218E/delH221 mutant	7LCA (chain A)	398	Sept 1st, 2021	97.36	97.35	96.78	96.72	96.71
Zoogloea ramigera	biosynthetic thiolase Q183Y mutant	7LCL (chain B)	399	Sept 1st, 2021	97.46	97.46	96.78	96.75	96.69
Homo sapiens	p97-D592N mutant bound to ADP	7RLG (chain A)	821	Sept 22nd, 2021	81.32	80.65	80.54	79.99	79.62
Homo sapiens	NPC1L1 mutant-W347R	7N4X	1332	Sept 1st, 2021	84.41	83.97	83.43	83.31	82.80

AlphaFold2 confident score: pLDDT; PDB: protein data bank.

Supplementary table 2: Structure similarity analysis of AlphaFold2 prediction on 10 experimentally determined variant protein structures from the protein data bank.

			Rank 0			Rank 1			Rank 2			Rank 3			Rank 4		
**PDB ID**	**Variant**	**Size (residues)**	**TM-score**	**lDDT**	**RMSD**	**TM-score**	**lDDT**	**RMSD**	**TM-score**	**lDDT**	**RMSD**	**TM-score**	**lDDT**	**RMSD**	**TM-score**	**lDDT**	**RMSD**
6SSO	p.L45H	135	0.997	0.956	0.24	0.997	0.954	0.24	0.991	0.936	0.41	0.992	0.931	0.39	0.989	0.932	0.47
7AHF (chain A)	p.L114E	156	0.956	0.431	1.03	0.959	0.431	1.18	0.961	0.434	0.98	0.956	0.434	1.08	0.961	0.435	1.07
7K77	p.I38T	197	0.972	0.915	0.49	0.972	0.912	0.51	0.969	0.888	0.59	0.980	0.897	0.73	0.973	0.903	0.49
7K1A (chain B)	p.C137I/p.I141W/p.M167L/p.F168Y	211	0.991	0.461	0.63	0.990	0.461	0.66	0.988	0.453	0.67	0.988	0.453	0.66	0.987	0.453	0.67
7JZ7 (chain A)	p.S48F	292	0.995	0.478	0.47	0.994	0.476	0.51	0.994	0.473	0.54	0.994	0.474	0.52	0.995	0.476	0.49
7S2N (chain A)	p.F337L	381	0.927	0.469	1.56	0.930	0.469	1.52	0.953	0.454	1.24	0.951	0.454	1.28	0.946	0.456	1.36
7LCA (chain A)	p.Y218E/p.221delH	398	0.997	0.244	0.43	0.997	0.244	0.42	0.994	0.239	0.58	0.994	0.239	0.61	0.993	0.238	0.64
7LCL (chain B)	p.Q183Y	399	0.998	0.243	0.38	0.998	0.243	0.37	0.992	0.234	0.65	0.994	0.235	0.6	0.994	0.235	0.6
7RLG (chain A)	p.D592N	821	0.768	0.124	3.39	0.729	0.123	2.67	0.723	0.121	2.76	0.757	0.124	2.07	0.678	0.120	3.23
7N4X	p.W347R	1332	0.977	0.856	1.37	0.979	0.854	1.26	0.977	0.850	1.35	0.975	0.853	1.44	0.975	0.847	1.43

The AlphaFold2-predicted structures were compared to the experimentally determined protein structures from the protein data bank (PDB). lDDT: local Distance Difference Test; RMSD: root mean square deviation; TM: template modeling.

Supplementary table 3: Confident score of AlphaFold2 prediction on myocilin wildtype and variant protein structures.

Wildtype/Variants	PDB ID	Residues	Rank 0	Rank 1	Rank 2	Rank 3	Rank 4
Wildtype	4WXQ	33-504	82.08 (27.65 – 98.72)	81.99 (26.56 – 98.77)	81.81 (24.31 – 98.88)	81.59 (31.57 – 98.66)	80.61 (26.24 – 98.72)
		33-201	72.66 (27.65 – 94.34)	73.11 (27.88 – 92.48)	70.94 (28.39 – 92.27)	73.56 (36.96 – 93.20)	70.39 (26.78 – 94.11)
		202-243	36.32 (29.45 – 48.75)	32.85 (26.56 – 45.36)	34.72 (24.31 – 53.24)	38.10 (31.57 – 70.16)	34.35 (26.24 – 63.57)
		244-504	95.55 (52.87 – 98.72)	95.65 (55.39 – 98.77)	96.43 (56.92 – 98.88)	93.79 (60.57 – 98.66)	94.67 (58.95 – 98.72)
p.E396D	4WXS	33-504	82.15 (27.87 – 98.72)	82.15 (22.24 – 98.81)	81.88 (27.20 – 98.71)	80.73 (31.09 – 98.65)	78.80 (26.25 – 98.59)
		33-201	73.37 (27.87 – 91.82)	71.79 (29.71 – 93.59)	71.65 (28.92 – 93.80)	71.29 (35.41 – 92.17)	65.68 (28.13 – 85.93)
		202-243	35.03 (27.87 – 51.96)	40.49 (22.24 – 55.42)	38.92 (27.20 – 52.00)	37.86 (31.09 – 70.75)	43.33 (26.25 – 62.98)
		244-504	95.42 (56.08 – 98.72)	95.56 (57.52 – 98.81)	95.41 (56.25 – 98.71)	93.74 (61.04 – 98.65)	93.00 (57.14 – 98.59)
p.D478N	6OU2	33-504	81.90 (28.16 – 98.72)	81.70 (24.47 – 98.84)	80.89 (27.49 – 98.72)	80.46 (30.65 – 98.60)	77.94 (25.04 – 98.58)
		33-201	72.32 (28.60 – 93.47)	71.09 (29.96 – 89.86)	70.18 (28.42 – 89.83)	71.02 (36.41 – 92.61)	67.19 (26.67 – 89.36)
		202-243	36.33 (28.16 – 51.65)	37.08 (24.47 – 53.00)	36.03 (27.49 – 51.45)	38.82 (30.65 – 69.74)	30.11 (25.04 – 65.05)
		244-504	95.43 (57.51 – 98.72)	95.75 (57.09 – 98.84)	95.05 (56.86 – 98.72)	93.28 (59.49 – 98.60)	92.60 (55.94 – 98.58)
p.D478S	6OU3	33-504	81.99 (24.63 – 98.82)	81.82 (28.15 – 98.63)	81.71 (24.83 – 98.76)	80.95 (29.44 – 98.52)	78.58 (25.27 – 98.65)
		33-201	72.14 (29.18 – 93.17)	72.36 (28.15 – 94.79)	72.54 (25.97 – 91.78)	72.43 (35.33 – 91.28)	66.49 (26.91 – 92.40)
		202-243	36.81 (24.63 – 50.79)	36.78 (28.26 – 47.68)	32.40 (24.83 – 49.17)	42.86 (29.44 – 65.19)	31.74 (25.27 – 67.53)
		244-504	95.63 (55.53 – 98.82)	95.19 (52.99 – 98.63)	95.59 (55.67 – 98.76)	92.59 (58.35 – 98.52)	93.94 (59.98 – 98.65)
p.D380A/p.D478S	6OU0	33-504	82.99 (26.99 – 98.82)	82.55 (28.72 – 98.70)	82.09 (27.56 – 98.66)	80.97 (27.84 – 98.75)	80.78 (28.36 – 98.79)
		33-201	72.98 (30.26 – 93.94)	74.18 (29.13 – 91.17)	72.68 (27.56 – 93.71)	68.03 (30.91 – 91.38)	70.39 (35.73 – 91.64)
		202-243	45.19 (26.99 – 63.41)	37.44 (28.72 – 51.42)	37.44 (28.07 – 48.55)	50.03 (27.84 – 76.89)	37.22 (28.36 – 72.40)
		244-504	95.55 (55.65 – 98.82)	95.22 (55.82 – 98.70)	95.38 (52.88 – 98.66)	94.32 (58.04 – 98.75)	94.52 (60.77 – 98.79)
p.N428D/p.D478H	6PKD	33-504	82.65 (25.86 – 98.86)	82.21 (26.81 – 98.76)	81.99 (27.05 – 98.78)	81.05 (30.11 – 98.56)	79.65 (26.51 – 98.69)
		33-201	73.04 (27.94 – 95.07)	72.22 (28.60 – 94.30)	72.43 (28.86 – 91.07)	72.48 (35.49 – 91.23)	68.20 (29.64 – 91.72)
		202-243	37.66 (25.86 – 53.42)	39.19 (26.81 – 53.11)	36.58 (27.05 – 52.08)	43.78 (30.11 – 67.29)	44.42 (26.51 – 67.41)
		244-504	96.10 (58.95 – 98.86)	95.60 (55.12 – 98.76)	95.49 (55.12 – 98.78)	92.61 (58.11 – 98.56)	92.73 (50.86 – 98.69)

p.N428E/p.D478K	6PKF	33-504	82.50 (27.50 – 98.81)	82.28 (25.48 – 98.85)	81.80 (28.21 – 98.76)	80.56 (28.67 – 98.57)	78.32 (26.39 – 98.58)
		33-201	73.99 (27.93 – 91.83)	71.81 (29.17 – 93.02)	71.76 (28.21 – 93.51)	70.98 (35.08 – 91.17)	67.16 (26.65 – 90.65)
		202-243	34.71 (27.50 – 45.70)	39.99 (25.48 – 54.89)	36.84 (29.25 – 49.75)	44.35 (28.67 – 62.70)	37.79 (26.39 – 63.56)
		244-504	95.70 (55.41 – 98.81)	95.87 (56.27 – 98.85)	95.53 (53.75 – 98.76)	92.59 (56.83 – 98.57)	92.07 (53.28 – 98.58)
p.N428E/p.D478S	6PKE	33-504	82.43 (28.60 – 98.80)	81.98 (26.66 – 98.85)	81.67 (27.70 – 98.80)	81.29 (27.07 – 98.77)	79.55 (26.03 – 98.84)
		33-201	72.97 (28.65 – 95.27)	71.46 (28.13 – 93.96)	72.40 (27.73 – 92.31)	73.00 (34.10 – 93.08)	68.34 (28.20 – 93.85)
		202-243	36.86 (28.60 – 50.06)	37.22 (26.66 – 54.07)	32.57 (27.70 – 45.23)	35.61 (27.07 – 71.69)	32.15 (26.03 – 69.29)
		244-504	95.89 (54.52 – 98.80)	95.99 (57.14 – 98.85)	95.58 (55.05 – 98.80)	94.01 (60.96 – 98.77)	94.44 (60.07 – 98.84)
p.Q48H	/	33-504	82.12 (28.16 – 98.72)	82.08 (27.20 – 98.74)	81.94 (24.70 – 98.87)	81.25 (29.26 – 98.68)	80.08 (25.62 – 98.60)
		33-201	73.08 (28.16 – 91.56)	71.82 (28.41 – 93.99)	71.08 (28.81 – 91.84)	73.31 (37.80 – 92.61)	68.68 (29.68 – 90.86)
		202-243	36.43 (28.71 – 48.48)	40.11 (27.20 – 53.74)	38.56 (24.70 – 52.25)	36.30 (29.26 – 70.00)	44.50 (25.62 – 69.20)
		244-504	95.32 (54.86 – 98.72)	95.47 (53.11 – 98.74)	95.95 (55.63 – 98.87)	93.63 (59.68 – 98.68)	93.19 (55.87 – 98.60)
p.D208E	/	33-504	81.96 (25.19 – 98.83)	81.86 (26.67 – 98.70)	81.09 (24.53 – 98.81)	80.67 (29.01 – 98.65)	80.19 (26.39 – 98.71)
		33-201	70.73 (29.36 – 92.66)	71.58 (29.00 – 93.42)	70.57 (29.41 – 91.64)	71.57 (32.68 – 91.77)	70.56 (26.39 – 91.97)
		202-243	43.10 (25.19 – 58.50)	41.00 (26.67 – 54.59)	32.19 (24.53 – 50.64)	37.25 (29.01 – 70.01)	34.93 (27.30 – 62.96)
		244-504	95.49 (55.66 – 98.83)	95.09 (52.05 – 98.70)	95.77 (56.30 – 98.81)	93.56 (58.89 – 98.65)	93.71 (58.35 – 98.71)
p.C245Y	/	33-504	82.09 (26.97 – 98.79)	81.80 (26.37 – 98.84)	81.78 (27.63 – 98.73)	80.40 (27.21 – 98.66)	79.01 (26.13 – 98.70)
		33-201	73.27 (28.24 – 92.31)	71.38 (28.65 – 93.08)	71.89 (27.63 – 94.63)	71.73 (35.49 – 92.89)	66.90 (26.69 – 90.57)
		202-243	33.93 (26.97 – 44.60)	40.09 (26.37 – 53.71)	37.55 (27.78 – 49.44)	35.39 (27.21 – 66.25)	34.59 (26.13 – 63.71)
		244-504	95.55 (51.01 – 98.79)	95.25 (48.55 – 98.84)	95.30 (52.01 – 98.73)	93.25 (58.91 – 98.66)	94.00 (58.45 – 98.70)
p.G252R	/	33-504	82.97 (27.74 – 98.82)	81.75 (27.14 – 98.74)	81.67 (27.10 – 98.70)	80.57 (30.10 – 98.64)	79.64 (26.11 – 98.61)
		33-201	71.85 (30.59 – 94.05)	72.29 (27.40 – 91.63)	71.69 (27.60 – 94.59)	71.60 (36.06 – 91.48)	68.81 (26.11 – 91.79)
		202-243	51.10 (27.74 – 76.71)	33.58 (27.14 – 51.78)	35.95 (27.10 – 51.04)	36.85 (30.10 – 68.28)	36.32 (26.79 – 60.45)
		244-504	95.30 (55.29 – 98.82)	95.63 (54.97 – 98.74)	95.49 (55.41 – 98.70)	93.41 (58.68 – 98.64)	93.63 (58.78 – 98.61)
p.S313F	/	33-504	82.45 (28.12 – 98.83)	82.12 (28.84 – 98.70)	81.63 (27.29 – 98.77)	80.55 (26.40 – 98.70)	79.43 (26.45 – 98.73)
		33-201	70.91 (31.95 – 92.97)	72.31 (28.84 – 94.45)	71.87 (27.29 – 91.57)	71.44 (31.41 – 91.91)	67.80 (26.45 – 91.59)
		202-243	48.75 (28.12 – 69.64)	40.38 (29.08 – 52.80)	34.73 (27.33 – 51.47)	35.62 (26.40 – 66.57)	35.84 (27.17 – 64.82)
		244-504	95.34 (56.19 – 98.83)	95.18 (52.86 – 98.70)	95.49 (56.82 – 98.77)	93.68 (59.90 – 98.70)	93.99 (59.57 – 98.73)

p.E323K	/	33-504	81.79 (26.30 – 98.67)	81.72 (26.51 – 98.70)	81.49 (24.14 – 98.77)	81.19 (30.75 – 98.67)	79.48 (24.48 – 98.78)
		33-201	71.82 (26.30 – 94.59)	72.47 (26.51 – 91.93)	70.53 (29.33 – 92.63)	72.93 (34.24 – 92.99)	68.01 (29.87 – 91.75)
		202-243	37.76 (28.60 – 47.68)	35.06 (28.44 – 45.18)	40.81 (24.14 – 57.00)	37.39 (30.75 – 70.15)	31.45 (24.48 – 67.45)
		244-504	95.33 (52.61 – 98.67)	95.21 (54.90 – 98.70)	95.13 (56.14 – 98.77)	93.58 (61.00 – 98.67)	94.64 (60.90 – 98.78)
p.T353I	/	33-504	82.64 (24.88 – 98.84)	82.30 (27.37 – 98.74)	82.08 (27.62 – 98.76)	81.03 (27.23 – 98.73)	80.50 (27.76 – 98.69)
		33-201	72.52 (28.38 – 93.78)	72.63 (27.37 – 94.37)	73.57 (27.62 – 92.08)	72.98 (35.38 – 92.48)	68.32 (31.74 – 91.86)
		202-243	42.39 (24.88 – 58.74)	39.77 (27.87 – 51.41)	33.01 (28.21 – 44.82)	34.58 (27.23 – 68.60)	47.88 (27.76 – 74.46)
		244-504	95.66 (55.00 – 98.84)	95.41 (52.37 – 98.74)	95.49 (55.56 – 98.76)	93.72 (58.56 – 98.73)	93.63 (58.08 – 98.69)
p.G367R	/	33-504	82.29 (28.11 – 98.84)	82.19 (26.99 – 98.70)	81.57 (26.59 – 98.71)	81.06 (29.30 – 98.69)	79.74 (25.86 – 98.76)
		33-201	72.09 (33.20 – 93.91)	73.09 (27.27 – 94.88)	72.35 (27.22 – 91.83)	72.47 (36.29 – 92.37)	68.69 (28.53 – 92.43)
		202-243	38.83 (28.11 – 53.38)	36.99 (26.99 – 51.47)	32.84 (26.59 – 53.56)	36.77 (29.30 – 69.85)	31.72 (25.86 – 68.79)
		244-504	95.89 (58.91 – 98.84)	95.35 (55.62 – 98.70)	95.38 (57.07 – 98.71)	93.74 (59.53 – 98.69)	94.62 (59.69 – 98.76)
p.Q368*	/	33-367	79.65 (30.84 – 98.25)	79.12 (30.25 – 98.31)	78.77 (29.92 – 98.26)	78.06 (32.72 – 98.19)	77.72 (27.99 – 97.97)
		33-201	78.84 (30.84 – 98.02)	77.81 (30.25 – 97.35)	78.25 (29.92 – 96.99)	76.72 (32.72 – 96.58)	77.43 (27.99 – 97.73)
		202-243	45.75 (34.95 – 60.96)	46.33 (33.01 – 59.10)	42.26 (30.14 – 57.73)	46.10 (34.80 – 78.41)	41.87 (29.75 – 78.89)
		244-367	92.25 (64.17 – 98.25)	92.01 (60.12 – 98.31)	91.86 (61.61 – 98.26)	90.71 (59.78 – 98.19)	90.26 (59.57 – 97.97)
p.P370L	/	33-504	82.03 (23.16 – 98.79)	82.00 (26.93 – 98.69)	81.55 (26.40 – 98.63)	80.70 (31.01 – 98.59)	80.36 (28.92 – 98.60)
		33-201	71.60 (29.53 – 93.18)	72.32 (27.65 – 94.81)	72.62 (26.40 – 92.54)	70.72 (33.42 – 91.75)	69.17 (31.45 – 92.08)
		202-243	40.47 (23.16 – 56.09)	38.77 (26.93 – 52.00)	33.10 (27.03 – 52.16)	45.64 (31.01 – 64.28)	46.23 (28.92 – 70.43)
		244-504	95.48 (56.95 – 98.79)	95.23 (56.18 – 98.69)	95.12 (56.16 – 98.63)	92.80 (57.21 – 98.59)	93.10 (52.65 – 98.60)
p.D384H	/	33-504	82.71 (27.49 – 98.70)	82.36 (28.70 – 98.74)	82.28 (26.88 – 98.86)	81.54 (30.10 – 98.72)	79.87 (23.83 – 98.81)
		33-201	74.25 (29.16 – 93.02)	72.30 (29.45 – 93.93)	70.73 (29.51 – 92.22)	73.12 (34.00 – 92.58)	68.48 (29.22 – 90.77)
		202-243	37.41 (27.49 – 50.47)	41.45 (28.70 – 56.19)	43.44 (26.88 – 59.31)	37.28 (30.10 – 68.76)	31.53 (23.83 – 66.57)
		244-504	95.47 (55.70 – 98.70)	95.45 (53.73 – 98.74)	96.02 (56.84 – 98.86)	94.12 (60.97 – 98.72)	95.03 (60.06 – 98.81)
p.A488V	/	33-504	82.24 (27.18 – 98.78)	81.93 (26.77 – 98.75)	81.54 (27.93 – 98.88)	81.40 (29.62 – 98.68)	80.11 (26.48 – 98.69)
		33-201	73.23 (28.56 – 93.25)	71.93 (26.77 – 94.47)	70.17 (27.93 – 91.49)	73.53 (38.35 – 92.37)	68.58 (28.20 – 90.43)
		202-243	35.32 (27.18 – 51.24)	36.39 (28.33 – 52.35)	35.43 (28.36 – 52.71)	36.87 (29.62 – 69.52)	43.21 (26.48 – 63.72)
		244-504	95.63 (56.00 – 98.78)	95.74 (56.88 – 98.75)	96.32 (56.39 – 98.88)	93.66 (58.79 – 98.68)	93.52 (56.06 – 98.69)

AlphaFold2 confident score (pLDDT) was presented in mean (range). PDB: protein data bank.

Supplementary table 4: Structure similarity analysis of AlphaFold2 prediction on the experimentally determined myocilin wildtype and variant protein structures from the protein data bank.

Wildtype/Variants	PDB ID	Rank 0			Rank 1			Rank 2			Rank 3			Rank 4		
		TM-score	lDDT	RMSD	TM-score	lDDT	RMSD	TM-score	lDDT	RMSD	TM-score	lDDT	RMSD	TM-score	lDDT	RMSD
Wildtype	4WXQ	0.980	0.911	0.88	0.979	0.909	0.89	0.980	0.908	0.84	0.979	0.901	0.86	0.980	0.904	0.84
p.E396D	4WXS	0.982	0.920	0.79	0.982	0.917	0.79	0.984	0.924	0.75	0.981	0.914	0.82	0.981	0.909	0.82
p.D478N	6OU2	0.940	0.843	1.58	0.939	0.841	1.59	0.941	0.848	1.57	0.953	0.873	1.36	0.954	0.870	1.35
p.D478S	6OU3	0.931	0.832	1.67	0.930	0.832	1.59	0.932	0.832	1.61	0.947	0.865	1.42	0.944	0.863	1.42
p.D380A/p.D478S	6OU0	0.939	0.835	1.60	0.941	0.841	1.58	0.940	0.836	1.59	0.949	0.863	1.40	0.951	0.866	1.39
p.N428D/p.D478H	6PKD	0.912	0.418	1.76	0.910	0.419	1.73	0.911	0.419	1.64	0.935	0.432	1.39	0.934	0.432	1.59
p.N428E/p.D478K	6PKF	0.899	0.799	1.42	0.897	0.800	1.44	0.898	0.799	1.45	0.912	0.834	1.29	0.913	0.834	1.28
p.N428E/p.D478S	6PKE	0.931	0.421	1.71	0.930	0.422	1.74	0.931	0.420	1.66	0.948	0.433	1.43	0.946	0.432	1.48

The AlphaFold2-predicted structures were compared to the experimentally determined protein structures from the protein data bank (PDB). lDDT: local Distance Difference Test; RMSD: root mean square deviation; TM: template modeling.

Supplementary table 5: Structure similarity analysis of the experimentally determined myocilin wildtype and variant protein structures from the protein data bank.

Variants	PDB ID	Residues	TM-score	lDDT	RMSD
p.E396D	4WXS	472	0.998	0.958	0.30
p.D478N	6OU2	472	0.906	0.779	1.46
p.D478S	6OU3	472	0.892	0.758	1.57
p.D380A/p.D478S	6OU0	472	0.891	0.754	1.51
p.N428D/p.D478H	6PKD	472	0.922	0.778	1.56
p.N428E/p.D478K	6PKF	472	0.902	0.770	1.41
p.N428E/p.D478S	6PKE	472	0.912	0.779	1.65

The experimentally determined myocilin variant structures were compared to the experimentally determined myocilin wildtype protein structure (4WXQ) from the protein data bank (PDB). lDDT: local Distance Difference Test; RMSD: root mean square deviation; TM: template modeling.

Supplementary table 6: Structure similarity analysis of the AlphaFold2-predicted full-length myocilin wildtype and variant protein structures with protein data bank identities.

Variants	PDB ID	Residues	Polyphen2	Rank	TM-score	lDDT	RMSD
p.E396D	4WXS	472	Probably damaging	Rank 2	0.782	0.914	2.23
p.D478N	6OU2	472	Probably damaging	Rank 4	0.598	0.876	0.99
p.D478S	6OU3	472	Possibly damaging	Rank 3	0.715	0.871	1.96
p.D380A/p.D478S	6OU0	472	Probably damaging	Rank 4	0.608	0.887	1.21
p.N428D/p.D478H	6PKD	472	Possibly damaging	Rank 3	0.697	0.868	1.96
p.N428E/p.D478K	6PKF	472	Probably damaging	Rank 4	0.679	0.890	1.71
p.N428E/p.D478S	6PKE	472	Probably damaging	Rank 3	0.610	0.853	1.03

The AlphaFold2-predicted myocilin variant structures (residue 33 – 504; with highest template modeling (TM)-score to the experimentally determined structures) with protein data bank (PDB) identities were compared to the AlphaFold2-predicted

myocilin wildtype protein structure (Rank 2; with highest TM-score to the experimentally determined structure). lDDT: local Distance Difference Test; Polyphen2: Polymorphism Phenotyping version 2; RMSD: root mean square deviation.

Supplementary table 7: Structure similarity analysis of the AlphaFold2-predicted full-length myocilin variant protein structures without experimentally determined structures.

			Rank 0			Rank 1			Rank 2			Rank 3			Rank 4		
**Variants**	**Residues**	**Polyphen2**	**TM-score**	**lDDT**	**RMSD**	**TM-score**	**lDDT**	**RMSD**	**TM-score**	**lDDT**	**RMSD**	**TM-score**	**lDDT**	**RMSD**	**TM-score**	**lDDT**	**RMSD**
p.Q48H	472	Benign	0.670	0.906	1.53	0.712	0.910	1.66	0.665	0.942	1.40	0.600	0.872	1.02	0.697	0.903	1.57
p.D208E	472	Probably damaging	0.695	0.931	1.68	0.733	0.909	1.55	0.727	0.917	1.63	0.606	0.889	1.02	0.645	0.900	1.38
p.C245Y	472	Probably damaging	0.769	0.901	2.16	0.650	0.928	1.30	0.728	0.901	1.85	0.598	0.887	0.94	0.628	0.915	1.33
p.G252R	472	Probably damaging	0.747	0.927	2.85	0.736	0.911	1.58	0.656	0.908	1.24	0.607	0.862	1.15	0.628	0.902	1.22
p.S313F	472	Probably damaging	0.759	0.941	1.70	0.776	0.903	1.89	0.662	0.909	1.52	0.649	0.880	1.35	0.648	0.884	1.52
p.E323K	472	Probably damaging	0.663	0.903	1.53	0.654	0.903	1.38	0.666	0.940	1.39	0.600	0.870	1.06	0.608	0.890	1.17
p.T353I	472	Possibly damaging	0.633	0.917	1.19	0.746	0.904	1.63	0.654	0.907	1.28	0.607	0.865	1.00	0.701	0.883	1.58
p.G367R	472	Probably damaging	0.605	0.930	0.69	0.795	0.911	2.03	0.622	0.892	1.10	0.612	0.864	0.88	0.593	0.898	0.85
p.Q368*	335	/	0.301	0.374	1.56	0.310	0.378	1.83	0.289	0.378	1.60	0.329	0.368	2.02	0.295	0.365	1.44
p.P370L	472	Probably damaging	0.659	0.936	2.87	0.734	0.915	1.76	0.653	0.911	1.41	0.744	0.873	1.99	0.718	0.883	1.62
p.D384H	472	Probably damaging	0.742	0.896	2.14	0.719	0.904	1.60	0.673	0.943	1.59	0.603	0.870	1.12	0.624	0.892	1.37
p.A488V	472	Probably damaging	0.701	0.901	1.89	0.661	0.915	1.50	0.661	0.948	1.14	0.602	0.862	1.06	0.682	0.898	1.78

The AlphaFold2-predicted myocilin variant structures (residue 33 – 504) without experimentally determined structures were compared to the AlphaFold2-predicted myocilin wildtype protein structure (Rank 2; with highest TM-score to the experimentally determined structure). lDDT: local Distance Difference Test; Polyphen2: Polymorphism Phenotyping version 2; RMSD: root mean square deviation; TM: template modeling.

Supplementary table 8: Molecular docking analysis on the experimentally determined myocilin wildtype and variant protein structures.

	**Site 1**		**Site 2**		**Site 3**		**Site 4**		**Site 5**	
4WXQ	Site score	1.07	Site score	0.83	Site score	0.79	Site score	0.90	Site score	0.60
Wildtype	Docking score	Interaction	Docking score	Interaction	Docking score	Interaction	Docking score	Interaction	Docking score	Interaction
Apigenin	-7.04	Y267, L322	-4.90	I459, K461	-5.15	E261, D294	Not binding	/	-5.21	K275, D384
Gw5074	-4.88	S324, Y376	-2.81	K461	-3.12	D294	Not binding	/	-0.43	K275
4WXS	Site score	0.88	Site score	0.79	Site score	0.71	Site score	0.93	Site score	0.68
p.E396D	Docking score	Interaction	Docking score	Interaction	Docking score	Interaction	Docking score	Interaction	Docking score	Interaction
Apigenin	-6.20	S324, E340, H366, Y376	-5.77	S444, S474	-4.97	E261, D294, F299	Not binding	/	-5.99	C433, I459, K461
Gw5074	-5.38	S324, H366, Y376	-3.94	W373, N469	-2.89	R296, Q297, D289	Not binding	/	-0.23	T435, K461, S502
6OU2	Site score	0.93	Site score	0.80	Site score	0.97	Site score	0.74	Site score	0.63
p.D478N	Docking score	Interaction	Docking score	Interaction	Docking score	Interaction	Docking score	Interaction	Docking score	Interaction
Apigenin	-5.55	E253, R470, D490	-5.56	L322, S474	Not binding	/	-4.89	L257, Y267, W270	-4.47	A445, D446, R470
Gw5074	-1.91	K468, H470	-3.27	S474	-2.16	D273	-2.16	Y267, W270	-1.99	N469, R470
6OU3	Site score	1.10	Site score	0.83	Site score	0.89	Site score	0.61	Site score	0.53
p.D478S	Docking score	Interaction	Docking score	Interaction	Docking score	Interaction	Docking score	Interaction	Docking score	Interaction
Apigenin	-5.15	R272, N480, F487	-5.20	L322, S474	Not binding	/	-3.83	/	-4.87	A386, P481
Gw5074	-1.72	K485	-3.44	/	Not binding	/	-2.39	/	-0.89	P274, Y330, K484
6OU0	Site score	0.94	Site score	0.95	Site score	0.90	Site score	0.64	Site score	0.71
p.D380A/p.D478S	Docking score	Interaction	Docking score	Interaction	Docking score	Interaction	Docking score	Interaction	Docking score	Interaction
Apigenin	-4.02	Y376	Not binding	/	Not binding	/	-4.10	W270, F307, M494	-5.00	C433, K461
Gw5074	-3.35	S474	-1.11	/	Not binding	/	-3.39	K266, Y267	-2.51	T435, K461

6PKD	Site score	1.10	Site score	0.74	Site score	0.59	Site score	0.58	Site score	0.67
p.N428D/p.D478H	Docking score	Interaction	Docking score	Interaction	Docking score	Interaction	Docking score	Interaction	Docking score	Interaction
Apigenin	-5.15	N480	-5.04	L322, Y376	-5.33	D242, K484, K485, K500	-4.89	T243, P274, K484	-4.50	D454
Gw5074	-1.47	R272	-2.66	T293	-2.38	K500	-3.38	K275, Y330, K484	-3.27	Q415
6PKF	Site score	1.05	Site score	0.93	Site score	0.75	Site score	0.60	Site score	0.64
p.N428E/p.D478K	Docking score	Interaction	Docking score	Interaction	Docking score	Interaction	Docking score	Interaction	Docking score	Interaction
Apigenin	-5.58	D294, Y376, Y473, S474	Not binding	/	-4.15	T259, R272, E483	-4.97	Y278, G332, R346	-4.47	C245
Gw5074	-3.24	Y376, W489	Not binding	/	-2.63	R258, T259	-2.64	G332, S333, R346	-2.91	T435, K461
6PKE	Site score	1.18	Site score	0.88	Site score	0.72	Site score	0.79	Site score	0.55
p.N428E/p.D478S	Docking score	Interaction	Docking score	Interaction	Docking score	Interaction	Docking score	Interaction	Docking score	Interaction
Apigenin	-4.42	N480, E483	-5.46	T293, Y376, N491	-5.44	L257, K472, S474	-4.59	R258, E483	-4.01	Y278, G332, N350
Gw5074	Not binding	/	-3.17	W489	-2.65	/	-2.22	R272, K485	-0.19	E278, E348, N350

Supplementary table 9: Molecular docking analysis on the AlphaFold2-predicted myocilin wildtype and variant protein structures.

	Site 1		Site 2		Site 3		Site 4		Site 5	
Wildtype	Site score	0.92	Site score	0.91	Site score	0.76	Site score	0.62	Site score	0.57
Rank 2	Docking score	Interaction	Docking score	Interaction	Docking score	Interaction	Docking score	Interaction	Docking score	Interaction
Apigenin	-6.65	D294, V295, E340, H366, Y376	-4.58	G239, K484	Not binding	/	/	/	-5.51	E230, S231
Gw5074	-5.68	L322, H366, Y376	-3.09	K484	Not binding	/	/	/	-3.22	K229, S233
p.E396D	Site score	0.99	Site score	0.81	Site score	0.74	Site score	0.92	Site score	/
Rank 2	Docking score	Interaction	Docking score	Interaction	Docking score	Interaction	Docking score	Interaction	Docking score	Interaction
Apigenin	-5.96	N420, S460	-4.29	G292, W373	-4.63	D242	Not binding	/	/	/
Gw5074	-3.75	N420	-4.00	Y267, Y376	-3.62	K503	Not binding	/	/	/
p.D478N	Site score	0.93	Site score	0.97	Site score	0.68	Site score	0.53	Site score	/
Rank 4	Docking score	Interaction	Docking score	Interaction	Docking score	Interaction	Docking score	Interaction	Docking score	Interaction
Apigenin	-5.82	D294, V295, E340, Y376	Not binding	/	-4.40	K461	-5.38	D242, E483, K485	/	/
Gw5074	-5.76	L322, Y376	Not binding	/	-2.14	K461	-2.66	D242, K500, K503	/	/
p.D478S	Site score	1.01	Site score	0.94	Site score	0.94	Site score	0.64	Site score	/
Rank 3	Docking score	Interaction	Docking score	Interaction	Docking score	Interaction	Docking score	Interaction	Docking score	Interaction
Apigenin	-5.13	S231, E247	-5.40	D294, V295	Not binding	/	-4.58	A397, E418, R422	/	/
Gw5074	-4.29	S233, N420	-5.35	L322, Y376	Not binding	/	-2.35	I227, R422	/	/
p.D380A/p.D478S	Site score	0.97	Site score	0.89	Site score	0.87	Site score	0.68	Site score	0.55
Rank 4	Docking score	Interaction	Docking score	Interaction	Docking score	Interaction	Docking score	Interaction	Docking score	Interaction
Apigenin	-5.11	D294, V295	Not binding	/	Not binding	/	-4.11	T457, I459, K503	-4.67	D289, V291
Gw5074	-5.69	L322, Y376	Not binding	/	Not binding	/	0.53	/	-2.60	R296, Q297
p.N428D/p.D478H	Site score	0.98	Site score	0.94	Site score	0.93	Site score	0.66	Site score	0.60
Rank 3	Docking score	Interaction	Docking score	Interaction	Docking score	Interaction	Docking score	Interaction	Docking score	Interaction
Apigenin	-4.97	S231, S233, E247, K461	-5.10	D294, V295	Not binding	/	-6.22	I227, N420, N450	-5.25	D242, K484, K485
Gw5074	-3.50	K229, S233	-5.23	L322, Y376	Not binding	/	-3.76	I227, S440, T448	-3.53	D242, K500, K503
p.N428E/p.D478K	Site score	0.92	Site score	0.94	Site score	0.87	Site score	0.93	Site score	0.62
Rank 4	Docking score	Interaction	Docking score	Interaction	Docking score	Interaction	Docking score	Interaction	Docking score	Interaction
Apigenin	-5.09	K229, S460	-5.78	D294	-4.59	K461, M504	Not binding	/	-5.29	D242, K484, K485
Gw5074	-2.55	I227, R422, S440	-5.40	L322, T325, Y376	-2.91	S233, L236, K461	Not binding	/	-2.96	K500, K503
p.N428E/p.D478S	Site score	0.94	Site score	0.90	Site score	0.71	Site score	/	Site score	/
Rank 3	Docking score	Interaction	Docking score	Interaction	Docking score	Interaction	Docking score	Interaction	Docking score	Interaction

Apigenin	-5.70	D294	Not binding	/	-4.63	I459, K461	/	/	/	/
Gw5074	-5.22	L322, Y376	Not binding	/	-1.84	/	/	/	/	/
p.C245Y	Site score	0.88	Site score	0.85	Site score	0.80	Site score	0.89	Site score	/
Rank 0	Docking score	Interaction	Docking score	Interaction	Docking score	Interaction	Docking score	Interaction	Docking score	Interaction
Apigenin	-4.85	K229, T462	-5.42	G292, W373	-4.94	Y437, K461	Not binding	/	/	/
Gw5074	-2.34	L228, F451	-4.18	Y267, Y376	-2.93	K405	Not binding	/	/	/
p.G252R	Site score	0.91	Site score	0.86	Site score	0.68	Site score	0.87	Site score	0.57
Rank 0	Docking score	Interaction	Docking score	Interaction	Docking score	Interaction	Docking score	Interaction	Docking score	Interaction
Apigenin	-7.07	D294, Y295, E340, Y376	-5.63	R237, K461, K503, M504	-6.09	D242, C245, I499	Not binding	/	-5.51	D289, V291
Gw5074	-4.69	L322, H366, Y376	-3.03	K461	-2.47	K500, L501	Not binding	/	-0.36	T293, D294, R296
p.S313F	Site score	0.96	Site score	0.94	Site score	0.88	Site score	/	Site score	/
Rank 0	Docking score	Interaction	Docking score	Interaction	Docking score	Interaction	Docking score	Interaction	Docking score	Interaction
Apigenin	-4.35	D454, K461	-7.18	D294, V295, E340, Y376	Not binding	/	/	/	/	/
Gw5074	-2.63	G234, R237, K461	-5.03	L322, H366, Y376	Not binding	/	/	/	/	/
p.E323K	Site score	0.98	Site score	0.93	Site score	0.68	Site score	0.58	Site score	/
Rank 0	Docking score	Interaction	Docking score	Interaction	Docking score	Interaction	Docking score	Interaction	Docking score	Interaction
Apigenin	-5.42	L236, K461	Not binding	/	-4.71	T290, G292, W373, Y376	-5.24	D289, V291	/	/
Gw5074	-3.26	G234, N420	Not binding	/	-2.56	K266, Y247, S324, Y376	-3.65	D294	/	/
p.T353I	Site score	0.99	Site score	0.81	Site score	0.91	Site score	0.58	Site score	/
Rank 1	Docking score	Interaction	Docking score	Interaction	Docking score	Interaction	Docking score	Interaction	Docking score	Interaction
Apigenin	-5.82	G234, L236, E247, T435	-5.95	G292, S324, W373	Not binding	/	-4.82	D289, V291	/	/
Gw5074	-4.38	G234, N420, T462	-3.12	E340, G375, Y376	Not binding	/	-3.66	D294	/	/
p.G367R	Site score	1.01	Site score	0.80	Site score	0.68	Site score	0.92	Site score	0.60
Rank 1	Docking score	Interaction	Docking score	Interaction	Docking score	Interaction	Docking score	Interaction	Docking score	Interaction
Apigenin	-5.38	N420, S460	-4.71	Y267, T290, E323	-5.31	G239, D242	Not binding	/	-4.87	D289, V291
Gw5074	-4.23	K461, T462	-4.91	Y267, T290, E340	-3.14	K503	Not binding	/	-2.24	R258, E261, T262, R287, F299
p.Q368*	Site score	0.84	Site score	0.66	Site score	0.55	Site score		Site score	/
Rank 0	Docking score	Interaction	Docking score	Interaction	Docking score	Interaction	Docking score	Interaction	Docking score	Interaction
Apigenin	-4.60	L255, L257, G265, W270	-5.18	S324, E340, H366	-4.93	D289, V291	/	/	/	/

Gw5074	-3.90	R258, K266	-3.95		-3.09	T264, D294	/	/	/	/
p.P370L	Site score	0.95	Site score	0.94	Site score	0.91	Site score	0.57	Site score	/
Rank 4	Docking score	Interaction	Docking score	Interaction	Docking score	Interaction	Docking score	Interaction	Docking score	Interaction
Apigenin	-6.02	D294, V295, E340, Y376	-5.32	M504	Not binding	/	-4.66	D289, V291	/	/
Gw5074	-5.64	L322, Y376	-2.89	L236, K461	Not binding	/	-2.57	R296, Q297	/	/
p.D384H	Site score	0.92	Site score	0.86	Site score	0.63	Site score	0.93	Site score	/
Rank 0	Docking score	Interaction	Docking score	Interaction	Docking score	Interaction	Docking score	Interaction	Docking score	Interaction
Apigenin	-5.45	D242, C433	-6.54	E340, H366, G375	-4.66	E230, S231, T462	Not binding	/	/	/
Gw5074	-3.48	C245, K503	-5.13	T290, Y267, G375, Y376	-2.57	S231, K461	Not binding	/	/	/
p.A488V	Site score	0.89	Site score	0.84	Site score	0.70	Site score	0.91	Site score	0.58
Rank 0	Docking score	Interaction	Docking score	Interaction	Docking score	Interaction	Docking score	Interaction	Docking score	Interaction
Apigenin	-6.56	E340, H366, Y376	-5.53	E240, D242, C433	-4.91	A397, G458	Not binding	/	-5.11	D289, V291
Gw5074	-4.32	Y376, Y473	-3.29	K503	-2.07	K398, E418, R422	Not binding	/	-3.33	D294

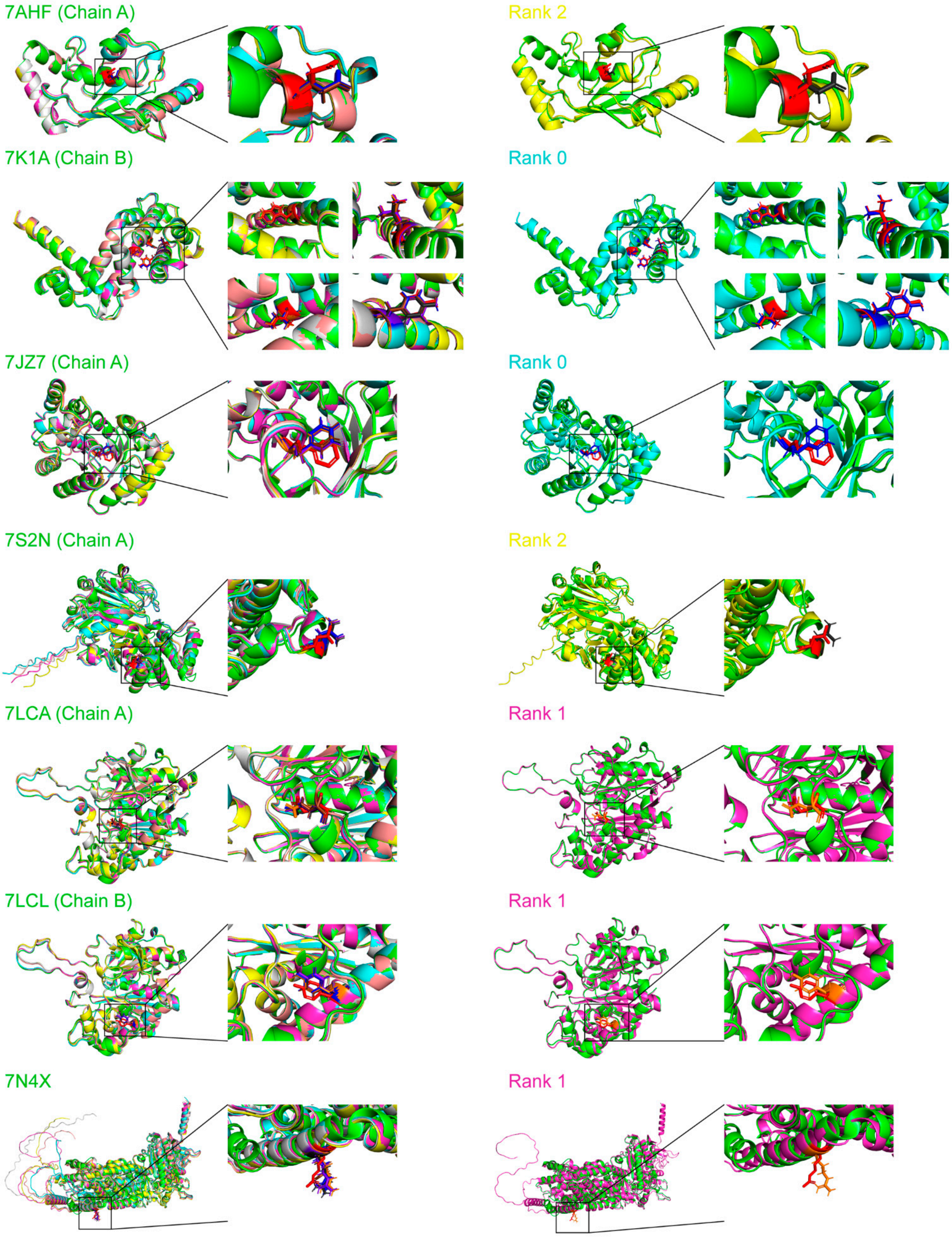
7AHF (Chain A)
Rank 2
7K1A (Chain B)
Rank 0
7JZ7 (Chain A)
Rank 0
7S2N (Chain A)
Rank 2
7LCA (Chain A)
Rank 1
7LCL (Chain B)
Rank 1
7N4X
Rank 1

Supplementary figure 1: Structure similarity analysis on the AlphaFold2-predicted variant protein structures to the experimentally determined structures from protein data bank.

The 5 AlphaFold2-predicted protein structures (Rank 0 – Rank 4) of variants from protein data bank (7AHF chain A, 7K1A chain B, 7JZ7 chain A, 7S2N chain A, 7LCA chain A, 7LCL chain B, and 7N4X) were aligned with the corresponding experimentally determined protein structures from protein data bank. The alignment of the AlphaFold2-predicted variant protein structures with highest template modeling score was shown in the right.

```
                     p.Q48H      p.D208E     p.C245Y p.G252R   p.S313F
Homo_sapiens         SGRCQYTFSV  DTLAFQELKS  GCGELVWVGE  QGYPSKVHIL
Pan_troglodytes      SGRCHYTFSV  DTLAFQELKS  GCGELVWVGE  QGYPSKVHIL
Bos_taurus           SGRCQYTFSV  ENMDFQELKS  GCGELVWVGE  QGYPSKVHVL
Rattus_norvegicus    SGRCQYTFTV  DTLAFQELKS  GCGVLMWVGE  QGYPSKVHVL
Mus_musculus         SGRCQYTFTV  DTLAFQELKS  GCGALVWVGE  QGYPSKVHVL
Gallus_gallus        SGRCTYSFTV  EPLGYQELKS  GCGELAWVGE  RGYPAKVHVL
Xenopus_tropicalis   SGQCTYSFTV  DPVGYQELKS  ACGELTWIGE  KGYPGKVYVL
Danio_rerio          NGRCQYTFMV  SNPGYQELTA  GCGDLVWVEN  RGFPTKVLLL
                      .*:* *:*   .   :***.:  .** * *: :  :*:* ** :*

                     p.E323K     p.T353I     p.G367R p.Q368* p.P370L   p.D380A p.D384H
Homo_sapiens         HILPRPLEST  LNTETVKAEK  GYHGQFPYSW  YTDIDLAVDE
Pan_troglodytes      HILPRPLEST  LNTETVKAEK  GYHGQFPYSW  YTDIDLAVDE
Bos_taurus           HVLPRPLEST  LRTETLKAEK  GYHGQFPYSW  YTDIDLAVDE
Rattus_norvegicus    HVLPQALEST  LNTETVKAEK  GYHGQFPYAW  YTDIDLAVDE
Mus_musculus         HVLPRALEST  LDTETVKAEK  GYHGHFPYAW  YTDIDLAVDE
Gallus_gallus        HVLPRPLEST  LRAEAISAER  GYHGQYPYSW  YTDIDLAVDE
Xenopus_tropicalis   YVLPRSMESN  FKTESVAVQR  GYQGQYPYSW  YTDIDLAVDE
Danio_rerio          LLLPESVEST  LHAESIAARR  GFHGQFPYSW  YTDIDLAIDE
                      :**. :**.  : :*:: ..:  *::*::**:*  *******:**

                     p.E396D     p.N428D/E   p.D478H/K/N/S   p.A488V
Homo_sapiens         YSTDEAKGAI  TWETNIRKQS  SSMIDYNPLE  KKLFAWDNLN
Pan_troglodytes      YSTDEAKGAI  TWETNIRKQS  SSMIDYNPLE  KKLFAWDNLN
Bos_taurus           YSTEAAKGAI  TWETNIRKQS  SSMIDYNPLE  RKLFAWDNFN
Rattus_norvegicus    YSTEENRGAI  TWETNIRKQS  SSMVDYNPLE  RKLFAWDNFN
Mus_musculus         YSTEEAKGAI  TWETNIRKQS  SSMIDYNPLE  RKLFAWDNFN
Gallus_gallus        YSTEKAKGAI  TWETNIRKQS  LSMVDYNPAE  RRLFAWDSYN
Xenopus_tropicalis   YSTEKAKGSI  SWETQIRKQS  ASMIDYNPTE  KKIYGWDNFN
Danio_rerio          YSTNKAKGAI  TWETKIRKTS  NSMVDYNSAK  RKLYAWDNYY
                     ***:  :*:*  :***:*** *   **:***  :  ::::.**.
```

Supplementary figure 2: Multiple sequence analysis on myocilin variant sites.

Multiple sequence analysis on 16 myocilin variant sites by Clustal Omega: p.Q48H, p.D208E, p.C245Y, p.G252R, p.S313F, p.E323K, p.T353I, p.G367R, p.Q368*, p.P370L p.D380A, p.D384H, p.E396D, p.N428D/E, p.D478H/K/N/S, and p.A488V.

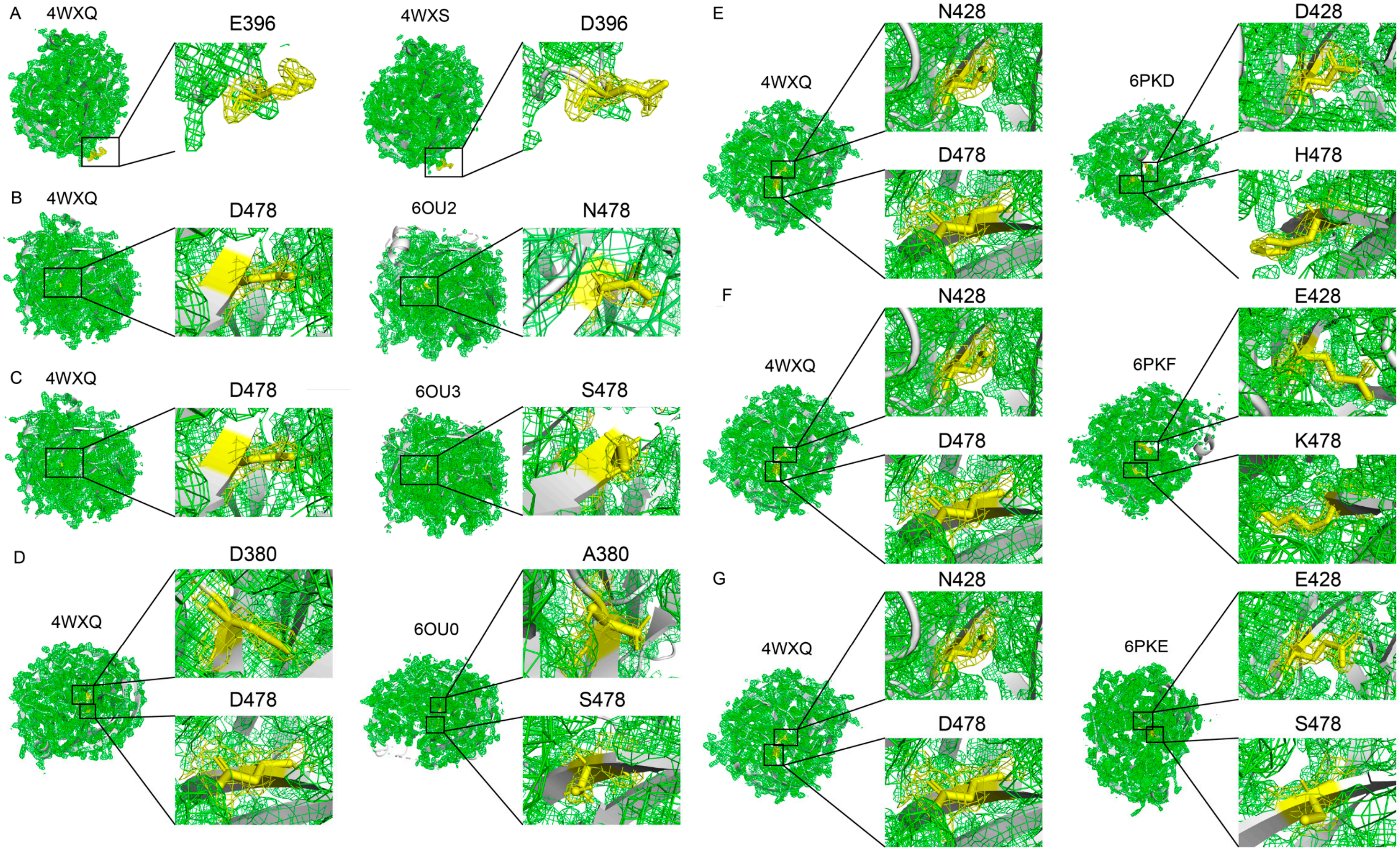
A
4WXQ
E396
4WXS
D396
B
4WXQ
D478
6OU2
N478
C
4WXQ
D478
6OU3
S478
D
4WXQ
D380
D478
6OU0
A380
S478
E
4WXQ
N428
D478
6PKD
D428
H478
F
4WXQ
N428
D478
6PKF
E428
K478
G
4WXQ
N428
D478
6PKE
E428
S478

Supplementary figure 3: Electron density maps of the experimentally determined myocilin wildtype and variant protein structures.

The electron density maps (green) of the experimentally determined myocilin wildtype and variants protein structures: (**A**) comparison between 4WXS and 4WXQ; (**B**) comparison between 6OU2 and 4WXQ; (**C**) comparison between 6OU3 and 4WXQ; (**D**) comparison between 6OU0 and 4WXQ; (**E**) comparison between 6PKD and 4WXQ; (**F**) comparison between 6PKF and 4WXQ; (**G**) comparison between 6PKE and 4WXQ. Yellow: the residues of amino acid changes and their electron density maps.

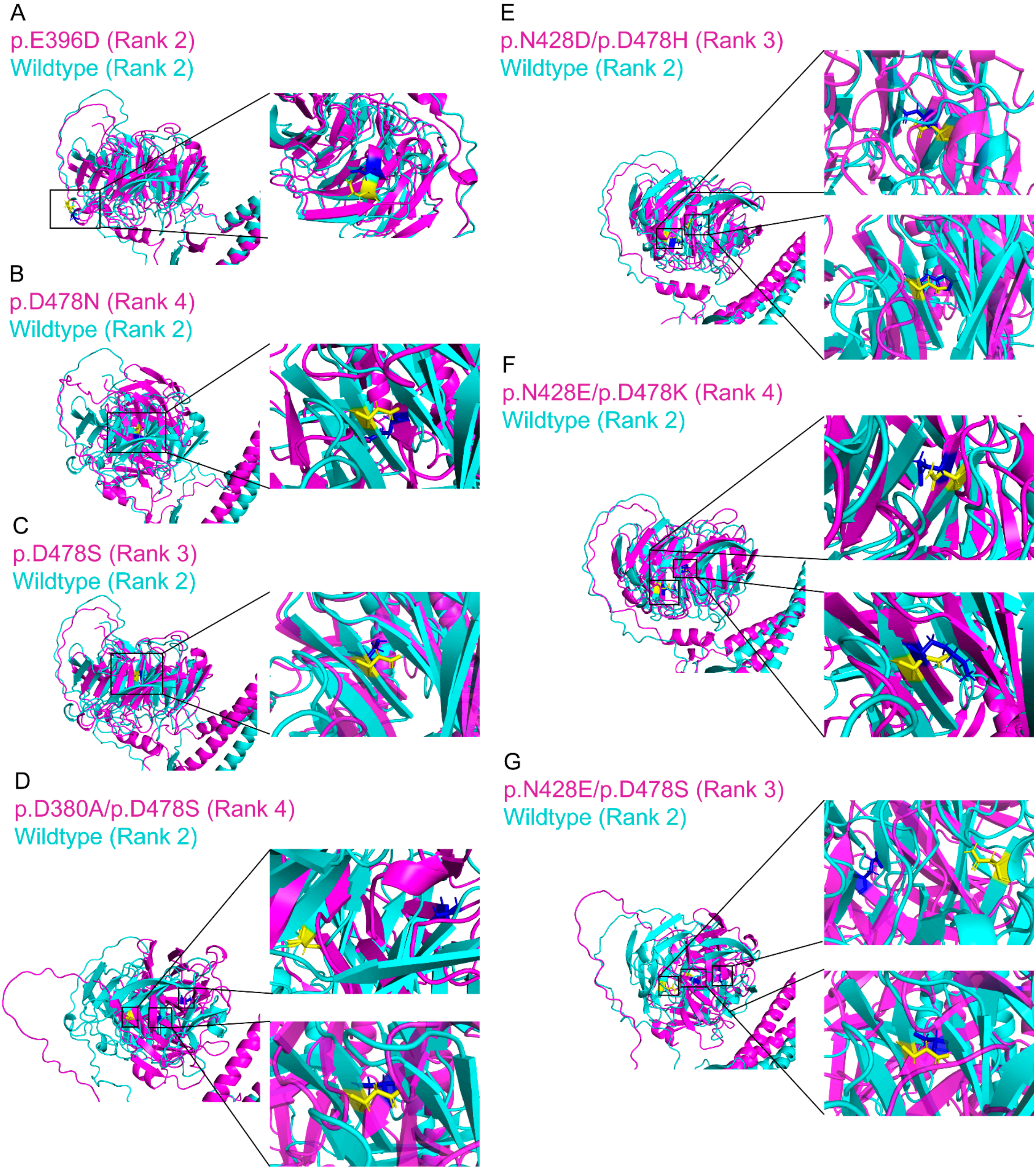
A
p.E396D (Rank 2)
Wildtype (Rank 2)
B
p.D478N (Rank 4)
Wildtype (Rank 2)
C
p.D478S (Rank 3)
Wildtype (Rank 2)
D
p.D380A/p.D478S (Rank 4)
Wildtype (Rank 2)
E
p.N428D/p.D478H (Rank 3)
Wildtype (Rank 2)
F
p.N428E/p.D478K (Rank 4)
Wildtype (Rank 2)
G
p.N428E/p.D478S (Rank 3)
Wildtype (Rank 2)

Supplementary figure 4: Structure similarity analysis of the AlphaFold2-predicted full-length myocilin wildtype and variant protein structures with protein data bank identities.

The AlphaFold2-predicted full-length protein structures of myocilin variants ((**A**) p.E396D; (**B**) p.D478N; (**C**) p.D478S; (**D**) p.D380A/p.D478S; (**E**) p.N428D/p.D478H; (**F**) p.N428E/p.D478K; (**G**) p.N428E/p.D478S) with protein data bank identities (red) were aligned and compared with the AlphaFold2-predicted myocilin wildtype protein structure (Rank 2) (green). Yellow: the side chain of the amino acid residue from the wildtype protein structure. Blue: the side chain of the amino acid residue from the variant protein structures.

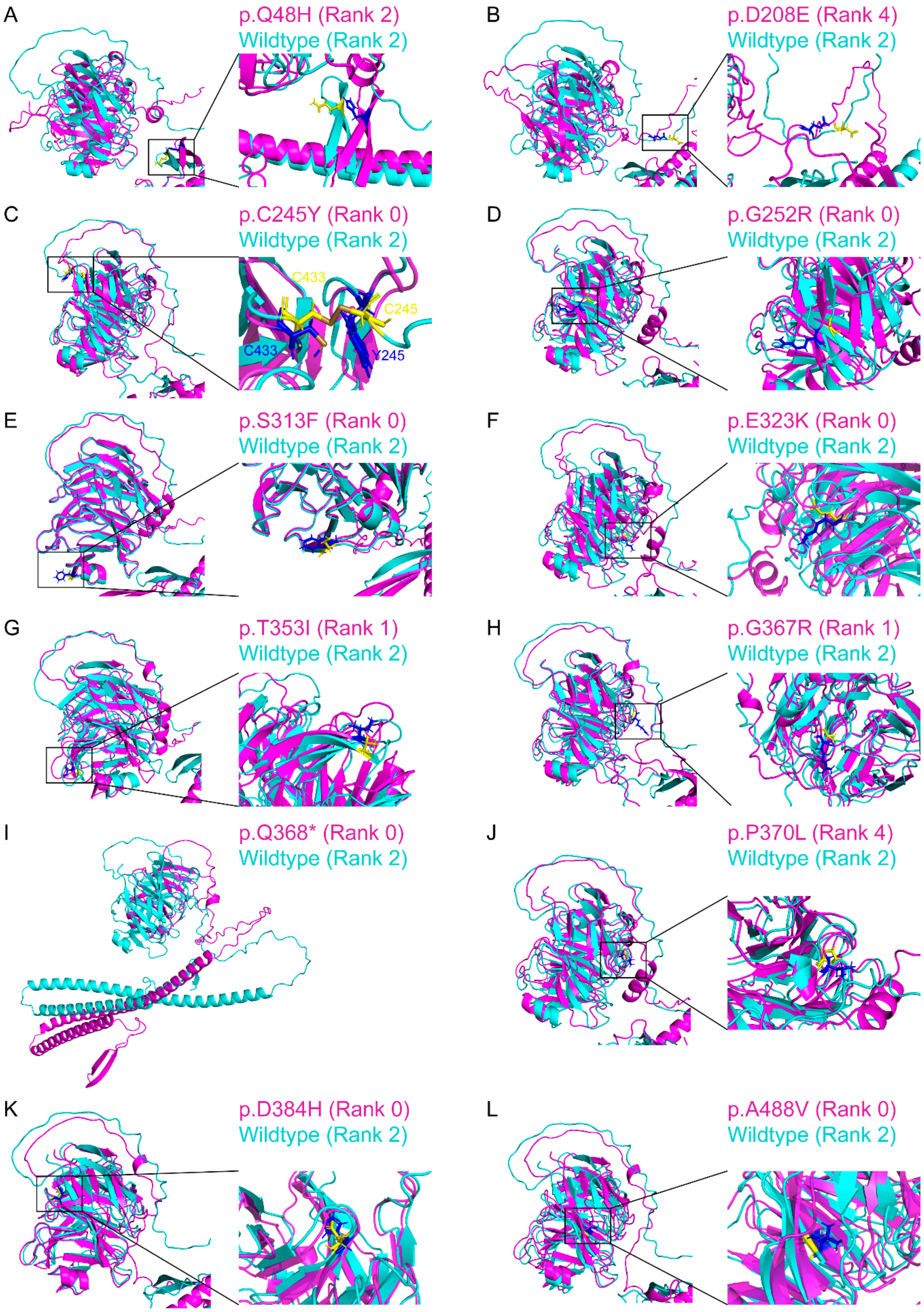
A
p.Q48H (Rank 2)
Wildtype (Rank 2)
B
p.D208E (Rank 4)
Wildtype (Rank 2)
C
p.C245Y (Rank 0)
Wildtype (Rank 2)
C433
C245
C433
Y245
D
p.G252R (Rank 0)
Wildtype (Rank 2)
E
p.S313F (Rank 0)
Wildtype (Rank 2)
F
p.E323K (Rank 0)
Wildtype (Rank 2)
G
p.T353I (Rank 1)
Wildtype (Rank 2)
H
p.G367R (Rank 1)
Wildtype (Rank 2)
I
p.Q368* (Rank 0)
Wildtype (Rank 2)
J
p.P370L (Rank 4)
Wildtype (Rank 2)
K
p.D384H (Rank 0)
Wildtype (Rank 2)
L
p.A488V (Rank 0)
Wildtype (Rank 2)

Supplementary figure 5: Structure similarity analysis of the AlphaFold2-predicted full-length structures of myocilin wildtype and variant proteins without experimentally determined structures.

The AlphaFold2-predicted full-length protein structures of myocilin variants ((**A**) p.C245Y; (**B**) p.G252R; (**C**) p.S313F; (**D**) p.E323K; (**E**) p.T353I; (**F**) p.G367R; (**G**) p.Q368*; (**H**) p.P370L; (**I**) p.D384H; (**J**) p.A488V) without experimentally determined structures (red) were aligned with the AlphaFold2-predicted myocilin wildtype protein structure (Rank 2) (green). Yellow: the side chain of the amino acid residue from the wildtype protein structure. Blue: the side chain of the amino acid residue from the variant protein structures. Brown: disulfide bond.

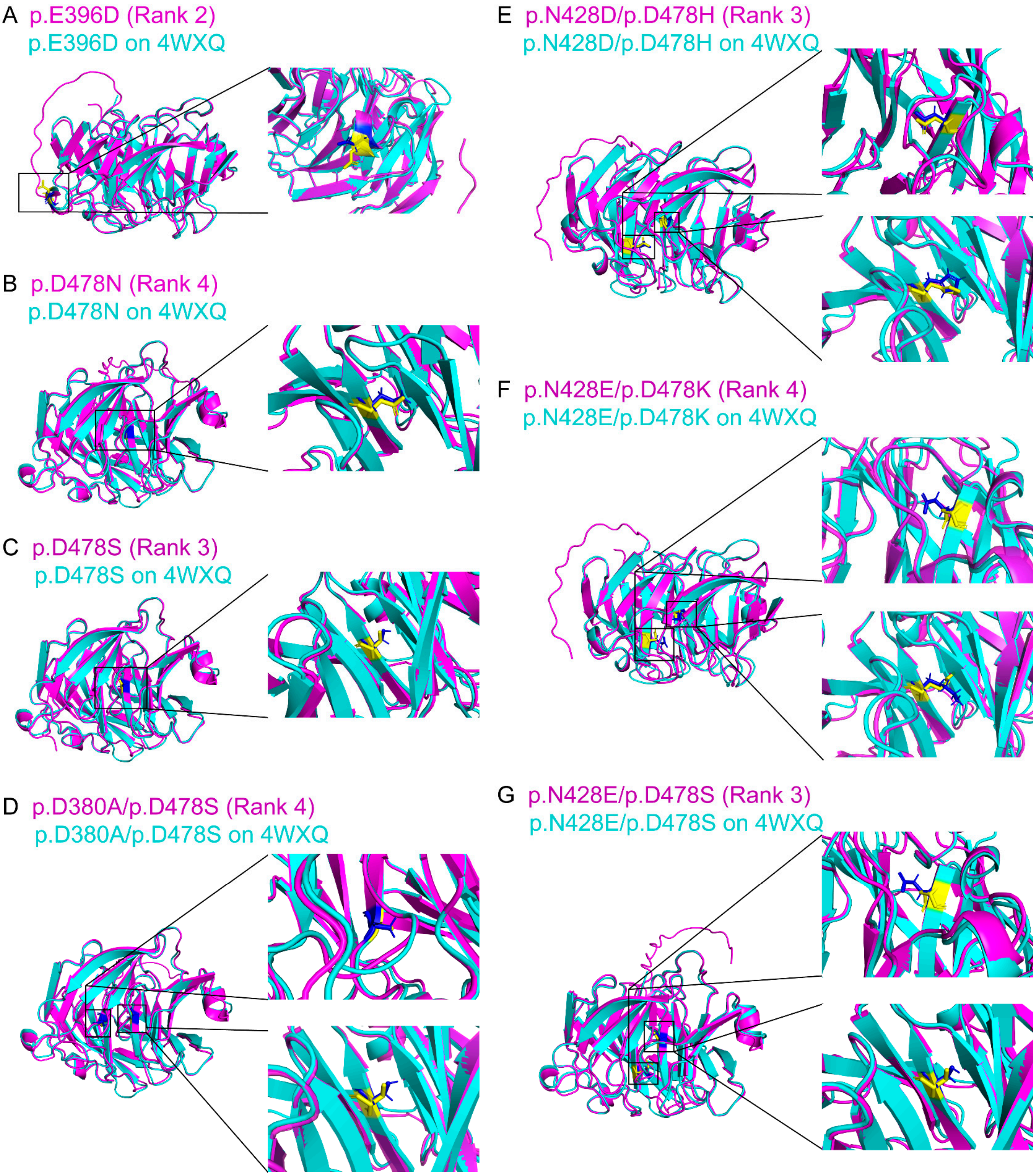
A p.E396D (Rank 2)
p.E396D on 4WXQ
B p.D478N (Rank 4)
p.D478N on 4WXQ
C p.D478S (Rank 3)
p.D478S on 4WXQ
D p.D380A/p.D478S (Rank 4)
p.D380A/p.D478S on 4WXQ
E p.N428D/p.D478H (Rank 3)
p.N428D/p.D478H on 4WXQ
F p.N428E/p.D478K (Rank 4)
p.N428E/p.D478K on 4WXQ
G p.N428E/p.D478S (Rank 3)
p.N428E/p.D478S on 4WXQ

Supplementary figure 6: Structure similarity analysis on the C-terminus of AlphaFold2-predicted myocilin variant protein structures with protein data bank identities to the amino acid substitution on the experimentally determined myocilin wildtype structure.

The AlphaFold2-predicted C-terminus protein structures of myocilin variants ((**A**) p.E396D; (**B**) p.D478N; (**C**) p.D478S; (**D**) p.D380A/p.D478S; (**E**) p.N428D/p.D478H; (**F**) p.N428E/p.D478K; (**G**) p.N428E/p.D478S) with protein data bank identities (red) were aligned with the experimentally determined myocilin wildtype protein structure (4WXQ) with the corresponding amino acid substitutions (green). Yellow: the side chain of the amino acid residue from the wildtype protein structure with the corresponding amino acid substitutions. Blue: the side chain of the amino acid residue from the variant protein structures.

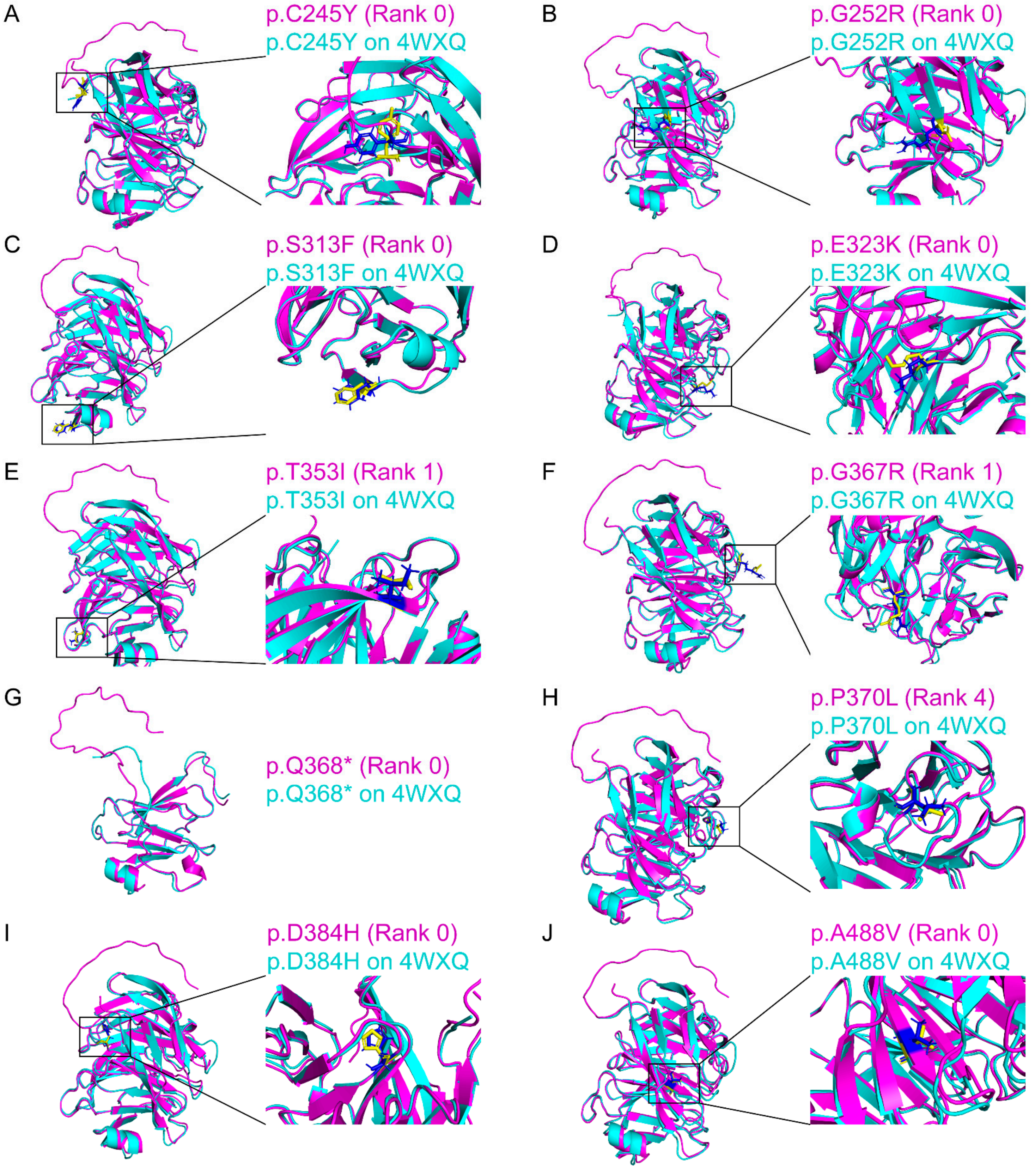
A
p.C245Y (Rank 0)
p.C245Y on 4WXQ
B
p.G252R (Rank 0)
p.G252R on 4WXQ
C
p.S313F (Rank 0)
p.S313F on 4WXQ
D
p.E323K (Rank 0)
p.E323K on 4WXQ
E
p.T353I (Rank 1)
p.T353I on 4WXQ
F
p.G367R (Rank 1)
p.G367R on 4WXQ
G
p.Q368* (Rank 0)
p.Q368* on 4WXQ
H
p.P370L (Rank 4)
p.P370L on 4WXQ
I
p.D384H (Rank 0)
p.D384H on 4WXQ
J
p.A488V (Rank 0)
p.A488V on 4WXQ

Supplementary figure 7: Structure similarity analysis on the C-terminus of AlphaFold2-predicted myocilin variant protein structures without previous experimental determination to the amino acid substitution on the experimentally determined myocilin wildtype structure.

The AlphaFold2-predicted C-terminus protein structures of myocilin variants ((**A**) p.C245Y; (**B**) p.G252R; (**C**) p.S313F; (**D**) p.E323K; (**E**) p.T353I; (**F**) p.G367R; (**G**) p.Q368*; (**H**) p.P370L; (**I**) p.D384H; (**J**) p.A488V) (red) were aligned with the experimentally determined myocilin wildtype protein structure (4WXQ) with the corresponding amino acid substitutions (green). Yellow: the side chain of the amino acid residue from the wildtype protein structure with the corresponding amino acid substitutions. Blue: the side chain of the amino acid residue from the variant protein structures.

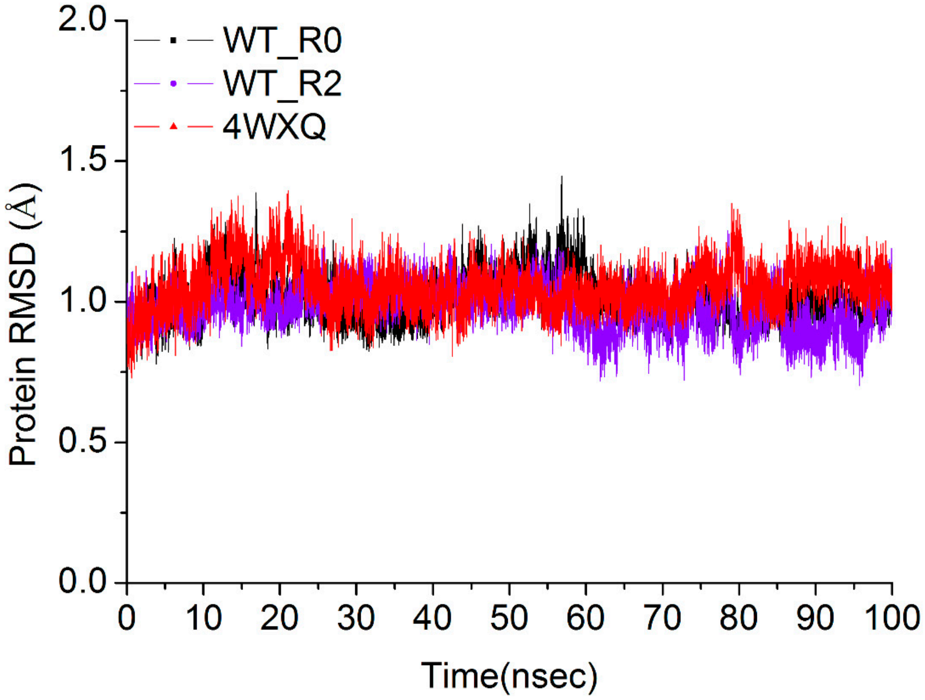

Supplementary figure 8: Root mean square deviation analysis of molecular dynamics on the experimentally determined and AlphaFold2-predicted wildtype myocilin protein structures.

Root mean square deviation (RMSD) comparisons among the olfactomedin-like domain of the AlphaFold2-predicted wildtype myocilin protein structures (Rank 0 and Rank 2) and the experimentally determined wildtype myocilin protein structure (4WXQ).

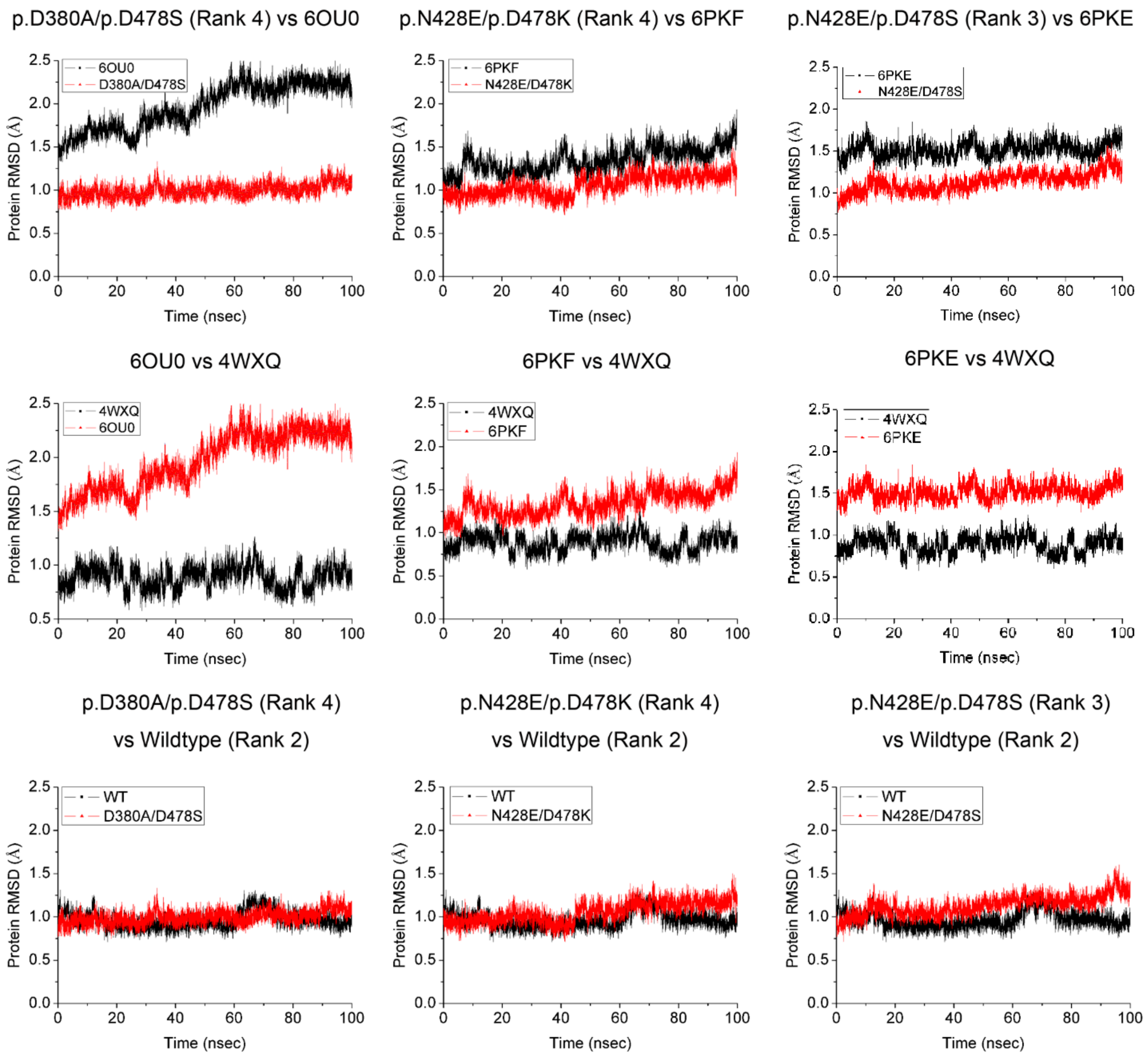

Supplementary figure 9: Root mean square deviation analysis of molecular dynamics on the experimentally determined and AlphaFold2-predicted myocilin wildtype and variant protein structures.

Root mean square deviation (RMSD) comparisons among the olfactomedin-like domain of the experimentally determined (6OU0, 6PKF, and 6PKE) and AlphaFold2-predicted myocilin variant protein structures (p.D380A/p.D478S (Rank 4), p.N428E/p.D478K (Rank 4), and p.N428E/p.D478S (Rank 3)) and the

experimentally determined (4WXQ) and AlphaFold2-predicted wildtype myocilin protein structure (Rank 2).

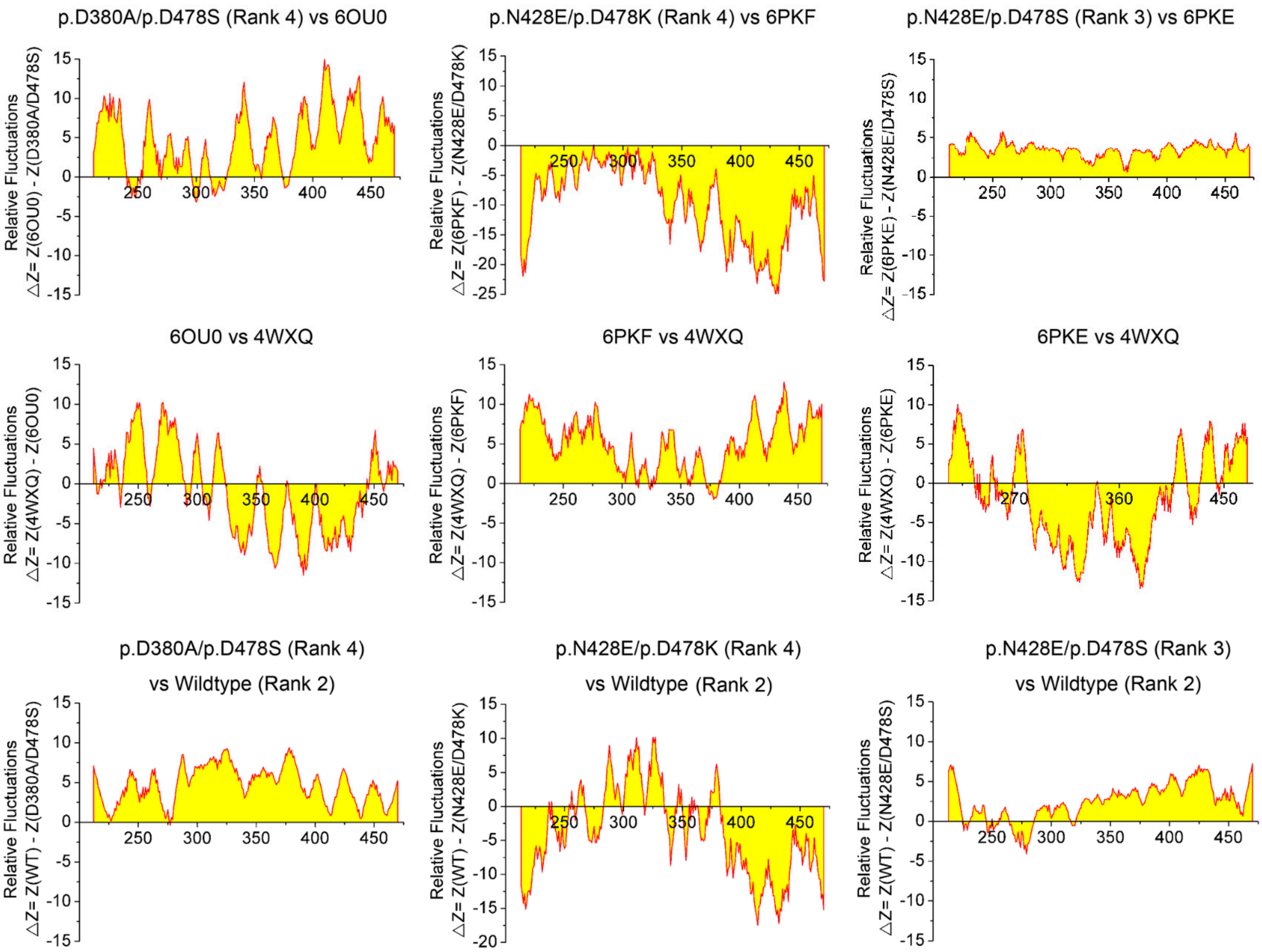

Supplementary figure 10: Root mean square fluctuation analysis of molecular dynamics on the experimentally determined and AlphaFold2-predicted myocilin wildtype and variant protein structures.

Root mean square fluctuation (RMSF) comparisons among the olfactomedin-like domain of the experimentally determined (6OU0, 6PKF, and 6PKE) and AlphaFold2-predicted myocilin variant protein structures (p.D380A/p.D478S (Rank 4), p.N428E/p.D478K (Rank 4), and p.N428E/p.D478S (Rank 3)) and the

experimentally determined (4WXQ) and AlphaFold2-predicted wildtype myocilin protein structure (Rank 2).

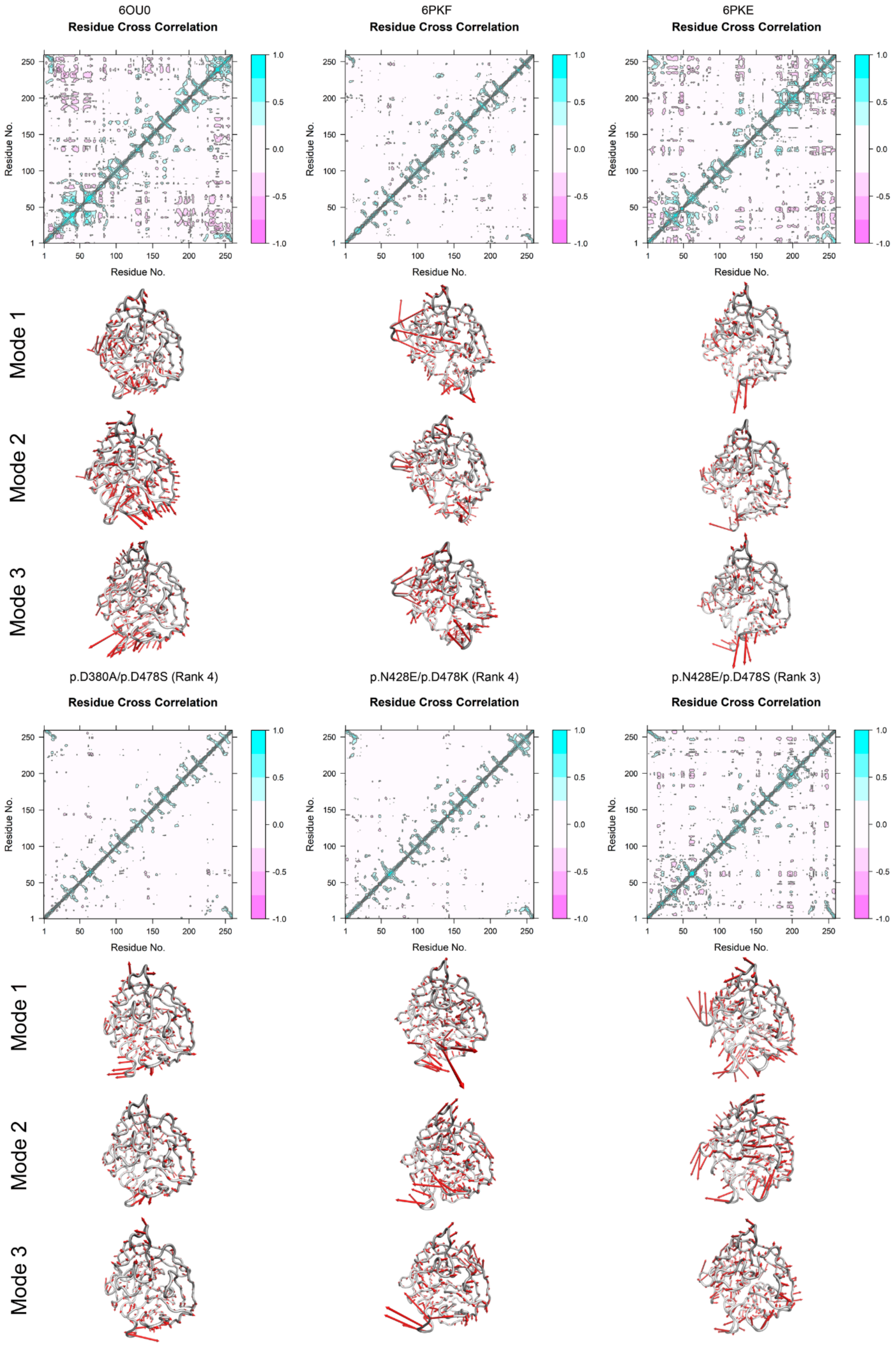
6OU0
Residue Cross Correlation
6PKF
Residue Cross Correlation
6PKE
Residue Cross Correlation
Residue No.
Mode 1
Mode 2
Mode 3
p.D380A/p.D478S (Rank 4)
Residue Cross Correlation
p.N428E/p.D478K (Rank 4)
Residue Cross Correlation
p.N428E/p.D478S (Rank 3)
Residue Cross Correlation
Residue No.
Mode 1
Mode 2
Mode 3

Supplementary figure 11: Residue cross-correlation and normal mode analyses of molecular dynamics on the experimentally determined and AlphaFold2-predicted myocilin variant protein structures.

Residue cross-correlation and normal mode analyses on the olfactomedin-like domain of the experimentally determined (6OU0, 6PKF, and 6PKE) and AlphaFold2-predicted myocilin variant protein structures (p.D380A/p.D478S (Rank 4), p.N428E/p.D478K (Rank 4), and p.N428E/p.D478S (Rank 3)) and the experimentally determined (4WXQ) and AlphaFold2-predicted wildtype myocilin protein structure (Rank 2).

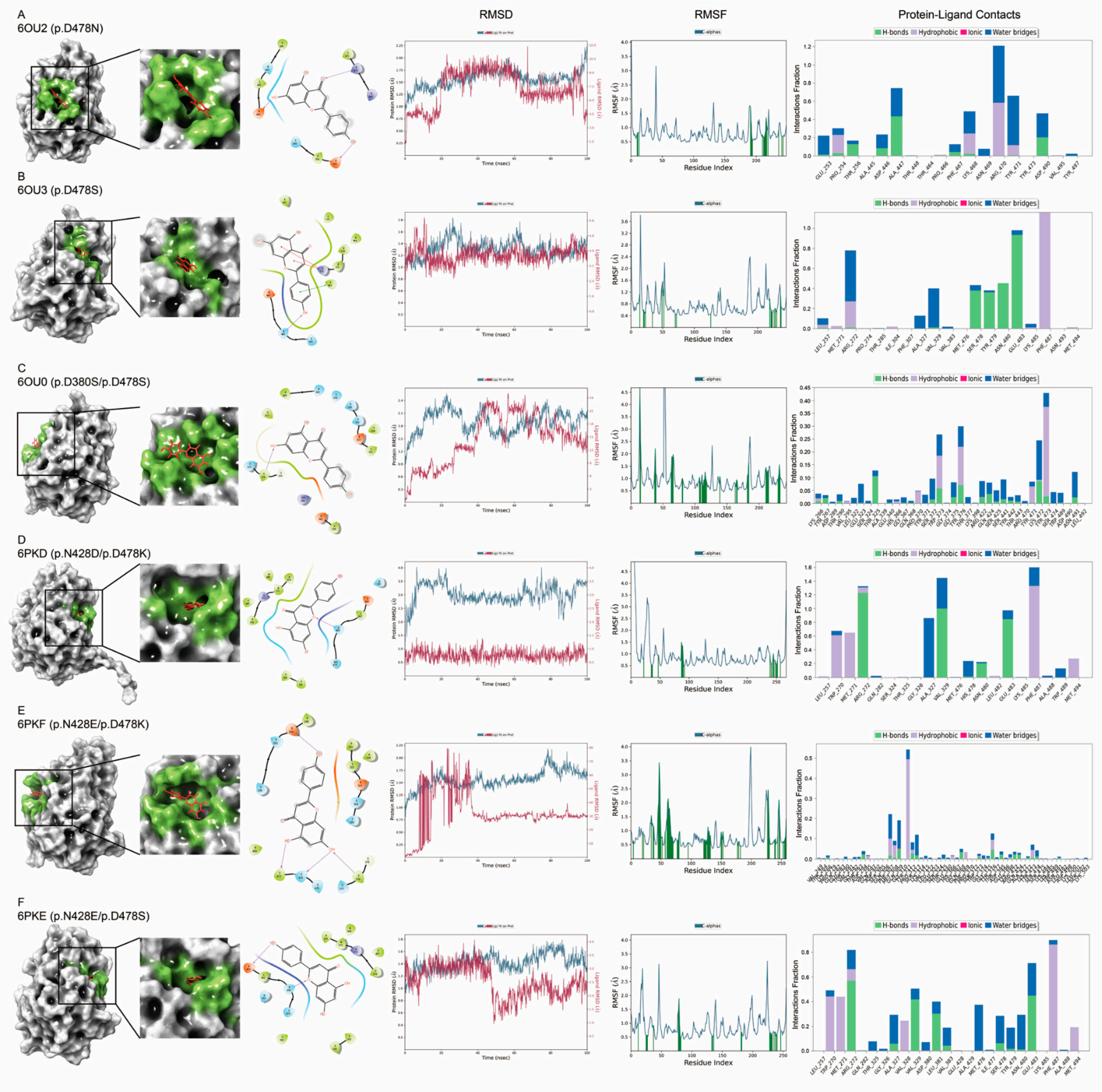
RMSD
RMSF
Protein-Ligand Contacts
A
6OU2 (p.D478N)
B
6OU3 (p.D478S)
C
6OU0 (p.D380S/p.D478S)
D
6PKD (p.N428D/p.D478K)
E
6PKF (p.N428E/p.D478K)
F
6PKE (p.N428E/p.D478S)
H-bonds
Hydrophobic
Ionic
Water bridges
Time (nsec)
RMSF (Å)
Residue Index
Interactions Fraction

Supplementary figure 12: Molecular docking analysis of apigenin on the experimentally determined myocilin variant protein structures.

Molecular docking analysis of apigenin (red) on the experimentally determined myocilin variant protein structures (6OU2, p.D478N; 6OU3, p.D478S; 6OU0, p.D380S/p.D478S; 6PKD, p.N428D/p.D478K; 6PKF, p.N428E/p.D478K; 6PKE, p.N428E/p.D478S). The surface structural representation, binding site (green), protein-ligand root mean square deviation (RMSD), protein root mean square fluctuation (RMSF), and protein-ligand contacts were shown.

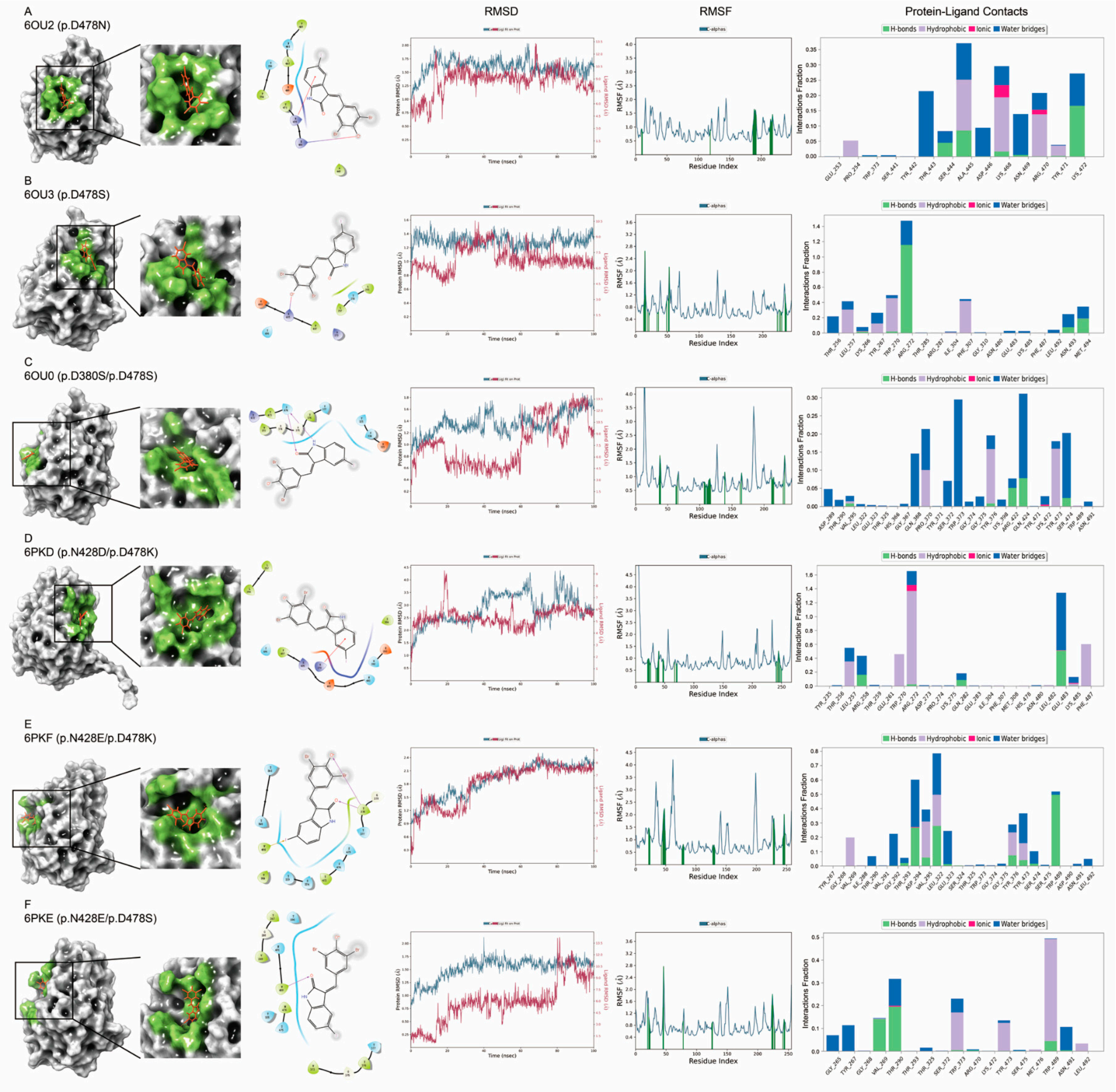
A
6OU2 (p.D478N)
B
6OU3 (p.D478S)
C
6OU0 (p.D380S/p.D478S)
D
6PKD (p.N428D/p.D478K)
E
6PKF (p.N428E/p.D478K)
F
6PKE (p.N428E/p.D478S)
RMSD
RMSF
Protein-Ligand Contacts
Time (nsec)
Residue Index
RMSF (Å)
Interactions Fraction
H-bonds
Hydrophobic
Ionic
Water bridges

Supplementary figure 13: Molecular docking analysis of Gw5074 on the experimentally determined myocilin variant protein structures.

Molecular docking analysis of Gw5074 (orange) on the experimentally determined myocilin variant protein structures (6OU2, p.D478N; 6OU3, p.D478S; 6OU0, p.D380S/p.D478S; 6PKD, p.N428D/p.D478K; 6PKF, p.N428E/p.D478K; 6PKE, p.N428E/p.D478S). The surface structural representation, binding site (green), protein-ligand root mean square deviation (RMSD), protein root mean square fluctuation (RMSF), and protein-ligand contacts were shown.

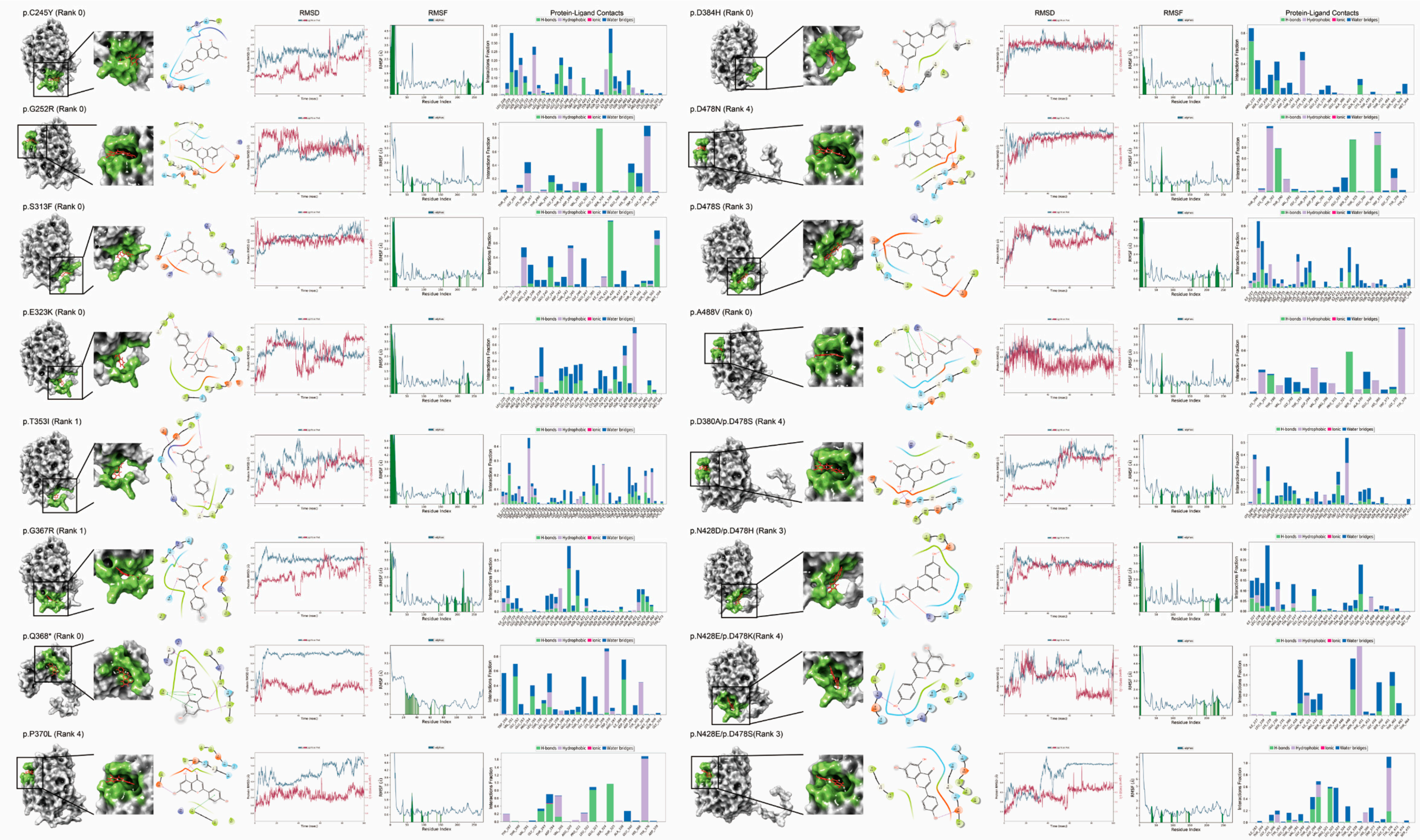
p.C245Y (Rank 0)
RMSD
RMSF
Protein-Ligand Contacts
p.G252R (Rank 0)
p.S313F (Rank 0)
p.E323K (Rank 0)
p.T353I (Rank 1)
p.G367R (Rank 1)
p.Q368* (Rank 0)
p.P370L (Rank 4)
p.D384H (Rank 0)
RMSD
RMSF
Protein-Ligand Contacts
p.D478N (Rank 4)
p.D478S (Rank 3)
p.A488V (Rank 0)
p.D380A/p.D478S (Rank 4)
p.N428D/p.D478H (Rank 3)
p.N428E/p.D478K(Rank 4)
p.N428E/p.D478S(Rank 3)
Residue Index
Interactions Fraction

Supplementary figure 14: Molecular docking analysis of apigenin on the AlphaFold2-predicted myocilin variant protein structures.

Molecular docking analysis of apigenin (red) on the AlphaFold2-predicted C-terminus protein structures of myocilin variants (p.C245Y Rank 0; p.G252R Rank 0; p.S313F Rank 0; p.E323K Rank 0; p.T353I Rank 1; p.G367R Rank 1; p.Q368* Rank 0; p.P370L Rank 4; p.D384H Rank 0; p.D478N Rank 4; p.D478S Rank 3; p.A488V Rank 0; p.D380A/p.D478S Rank 4; p.N428D/p.D478H Rank 3; p.N428E/p.D478K Rank 4; p.N428E/p.D478S Rank 3). The surface structural representation, binding site (green), protein-ligand root mean square deviation (RMSD), protein root mean square fluctuation (RMSF), and protein-ligand contacts were shown.

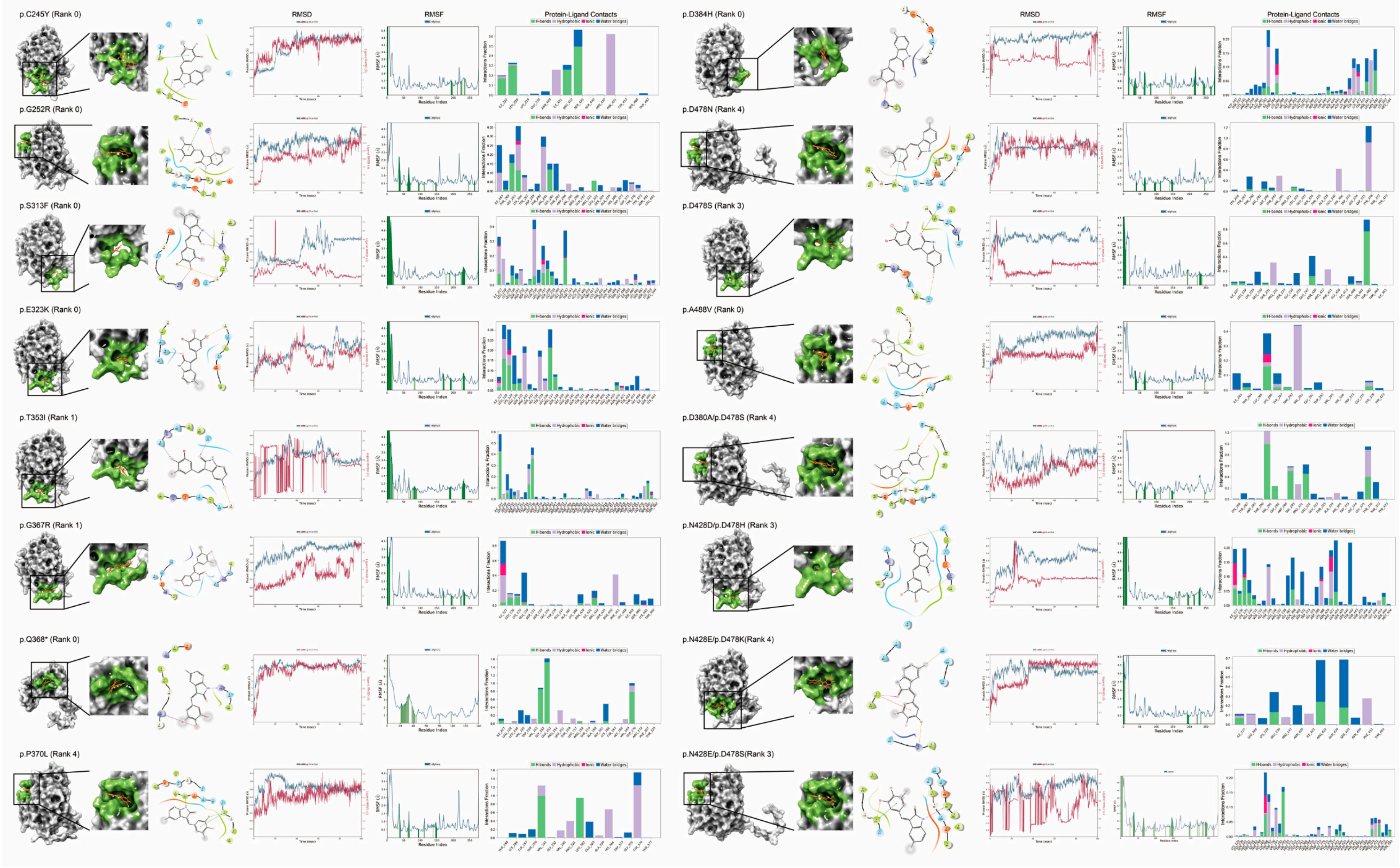
p.C245Y (Rank 0)
RMSD
RMSF
Protein-Ligand Contacts
H-bonds Hydrophobic Ionic Water bridges
Residue Index
Interactions Fraction
p.G252R (Rank 0)
p.S313F (Rank 0)
p.E323K (Rank 0)
p.T353I (Rank 1)
p.G367R (Rank 1)
p.Q368* (Rank 0)
p.P370L (Rank 4)
p.D384H (Rank 0)
RMSD
RMSF
Protein-Ligand Contacts
p.D478N (Rank 4)
p.D478S (Rank 3)
p.A488V (Rank 0)
p.D380A/p.D478S (Rank 4)
p.N428D/p.D478H (Rank 3)
p.N428E/p.D478K(Rank 4)
p.N428E/p.D478S(Rank 3)

Supplementary figure 15: Molecular docking analysis of Gw5074 on the AlphaFold2-predicted myocilin variant protein structures.

Molecular docking analysis of Gw5074 (orange) on the AlphaFold2-predicted C-terminus protein structures of myocilin variants (p.C245Y Rank 0; p.G252R Rank 0; p.S313F Rank 0; p.E323K Rank 0; p.T353I Rank 1; p.G367R Rank 1; p.Q368* Rank 0; p.P370L Rank 4; p.D384H Rank 0; p.D478N Rank 4; p.D478S Rank 3; p.A488V Rank 0; p.D380A/p.D478S Rank 4; p.N428D/p.D478H Rank 3; p.N428E/p.D478K Rank 4; p.N428E/p.D478S Rank 3). The surface structural representation, binding site (green), protein-ligand root mean square deviation (RMSD), protein root mean square fluctuation (RMSF), and protein-ligand contacts were shown.
